# Powering the Future: Opportunities and Obstacles in Lead‐Halide Inorganic Perovskite Solar Cells

**DOI:** 10.1002/advs.202412666

**Published:** 2025-02-03

**Authors:** Narendra Pai, Dechan Angmo

**Affiliations:** ^1^ Flexible Electronics Laboratory CSIRO Manufacturing Clayton VIC 3168 Australia

**Keywords:** CsPbX_3_, inorganic perovskite, ISOS, scalable, stability

## Abstract

Efficiency, stability, and cost are crucial considerations in the development of photovoltaic technology for commercialization. Perovskite solar cells (PSCs) are a promising third‐generation photovoltaic technology due to their high efficiency and low‐cost potential. However, the stability of organohalide perovskites remains a significant challenge. Inorganic perovskites, based on CsPbX_₃_ (X = Br^−^/I^−^), have garnered attention for their excellent thermal stability and optoelectronic properties comparable to those of organohalide perovskites. Nevertheless, the development of inorganic perovskites faces several hurdles, including the need for high‐temperature annealing to achieve the photoactive α‐phase and their susceptibility to transitioning into the nonphotoactive δ‐phase under environmental stressors, particularly moisture. These challenges impede the creation of high‐efficiency, high‐stability devices using low‐cost, scalable manufacturing processes. This review provides a comprehensive background on the fundamental structural, physical, and optoelectronic properties of inorganic lead‐halide perovskites. It discusses the latest advancements in fabricating inorganic PSCs at lower temperatures and under ambient conditions. Furthermore, it highlights the progress in state‐of‐the‐art inorganic devices, particularly those manufactured in ambient environments and at reduced temperatures, alongside simultaneous advancements in the upscaling and stability of inorganic PSCs.

## Graphical abstract

This review explores recent advancements, challenges, and future opportunities in the low‐cost fabrication of efficient inorganic perovskite solar cells (PSCs). It focuses on the manufacturing of PSCs under ambient conditions using low‐temperature processes. It examines the impact of moisture, oxygen, and temperature on perovskite film quality, emphasizing the importance of controlling these factors for high efficiency and scalability.

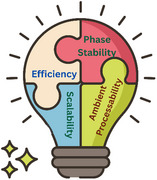

## 1 Introduction

The expeditious commercialization and adoption of photovoltaic technologies are paramount for transitioning toward a net zero scenario. Organic–inorganic hybrid perovskite solar cells (PSCs) demonstrate remarkable potential in this regard. A record power conversion efficiency (PCE) of 26.7% was recently reported for a single‐junction PSC. This efficiency is comparable with mature technologies such as single‐junction Si, CIGS, and CdTe solar cells.^[^ ^1^, ^2^, ^3^ ^]^ The high light‐absorption coefficient of PSCs enables optimal photovoltaic (PV) performance with a sub‐micrometer light‐absorbing layer thickness, unlike hundreds of micrometers required in conventional solar cells. Additionally, their solution processability makes them compatible with scalable deposition through vacuum‐free roll‐to‐roll printing and coating on low‐cost, flexible substrates. The combination of these advantages has the potential to significantly reduce material and manufacturing costs, making PV manufacturing more accessible than conventional Si‐based solar cells, which incur high capital costs, high energy‐intensive processing steps, and slow production throughput. Furthermore, PSC materials allow for tunability of their bandgap (*E*_g_), conduction and valence band energy levels, and absorption coefficients, enhancing their suitability for a wide range of applications in single‐junction and tandem structures.^[^ ^4^, ^5^, ^6^ ^]^

**Figure 1:**
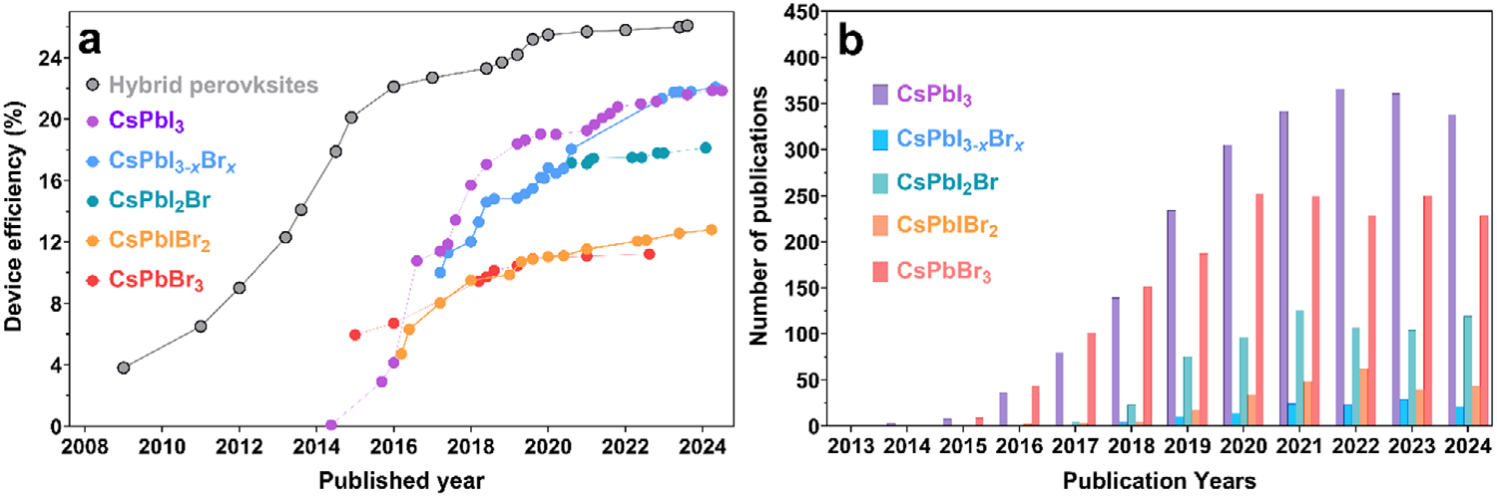
a) Best performance of inorganic Pb‐halide perovskites with respect to the reported year. The efficiencies of hybrid PSCs are included for comparison. b) Recorded number of articles on each topic with respect to the publication years are derived from Clarivate Web of Science. Copyright 2024. All rights reserved.

The unique properties of perovskites result from a combination of organic and inorganic components. However, the hygroscopicity and volatility of organic components^[^^]^ are associated with device stability challenges in PSCs. The hygroscopicity of alkylammonium organic cations results in the significant absorption of water, forming hydrated alkylammonium species that initiate perovskite degradation during long‐term operation and storage.^[^ ^11^, ^12^, ^13^ ^]^ The realization of stability challenges of organic–inorganic hybrid halide perovskite formulations and the potential of inorganic PSCs to overcome the issues have led to increased research on the latter. As a result, the record PCE for inorganic perovskites has surged from 0.09% to 22.1% in just nine years (**Figure** **1**a)^[^ ^14^, ^15^, ^16^, ^17^, ^18^, ^19^, ^20^, ^21^, ^22^ ^]^ and an increasing number of scholarly manuscripts have been published in the field, from three in 2014 to over 400 in 2024 (Figure 1b). An inorganic perovskite where organic cations are substituted with Cs^+^ effectively preserves the AMX_3_ perovskite structure. Particularly, inorganic CsPbX_3_ perovskite (where X = I^−^, Br^−^, or mixed‐halide) has gained significant traction owing to its remarkable optoelectronic properties, stability, and capacity to adopt a stable cubic perovskite phase, attributed to its close‐to‐desired tolerance factor of over 0.8.^[^ ^17^, ^23^, ^24^, ^25^ ^]^ The carrier lifetimes (≈50 ns), low excitonic binding energies (<100 meV), and high photoluminescence quantum yields (>70%) of inorganic perovskite allow for high optoelectronic properties.^[^ ^23^, ^26^, ^27^, ^28^, ^29^ ^]^

Inorganic Pb‐based perovskites for solar cells can be formed using CsPbX_3_, where X varies from I to Br. The bandgap of CsPbX_3_ depends on the relative composition of I to Br, ranging between 1.7 eV for CsPbI_3_ and 2.3 eV for CsPbBr_3_,^[^ ^30^, ^31^ ^]^ revealing the potential of inorganic perovskite materials to be incorporated into tandem solar cell applications. Despite their higher stability, chlorine and fluorine‐based inorganic perovskites (CsPbCl_3_ and CsPbF_3_) are not preferred because of their wide bandgap (>3 eV).^[^ ^32^, ^33^, ^34^ ^]^ Conversely, CsPbBr_3_ has attracted research interest due to its higher tolerance factor (0.86), resulting in exceptional thermal stability compared to its iodide counterparts.^[^ ^25^, ^35^, ^36^ ^]^ However, a wide bandgap of ≈2.3 eV implies significantly reduced light absorption (cut‐off at 540 nm), resulting in a theoretical PCE limit of 16.5%, of which 11.21% has been realized.^[^ ^37^, ^38^ ^]^ In contrast, iodide counterparts have a theoretical PCE limit of 28%, making them attractive for mass‐energy conversion applications.^[^ ^39^ ^]^ Notably, inorganic perovskites can endure extremely high temperatures of >500 °C without any degradation.^[^ ^40^, ^41^, ^42^ ^]^ However, phase stability at lower temperatures remains a bottleneck. The photoactive black cubic phase of CsPbI_3_ is only achievable at higher temperatures (>300 °C), as it transforms to a nonphotoactive δ‐yellow phase at lower temperatures (<180 °C) due to the lower borderline Goldschmidt's tolerance factor and the presence of humidity further accelerates this phase transformation.^[^ ^43^, ^44^, ^45^ ^]^

Research into solving the phase stability challenges associated with CsPbI_3_ has been bifurcated into two approaches. The first approach utilizes mixed‐halide inorganic perovskite materials for PSC devices despite increased bandgap. The cubic phase with monobromide substitution (CsPbI_2_Br) exhibits a higher *E*_g_ of 1.91 eV; however, remarkable long‐term device stability is achieved compared to its iodide counterparts along with a reasonable efficiency of 17.8%, with the α‐phase.^[^ ^46^ ^]^ Increasing the Br concentration can partially alleviate issues related to the phase segregation of the CsPbI_2_Br perovskite phase and its conversion to a non‐perovskite orthorhombic phase due to ambient moisture. However, researchers still face challenges owing to the limited solubility of Br precursors and their wider bandgap compared to their iodide counterparts. The second approach addresses the challenges of formulation engineering using additives and solvent modifications. Research has revealed that the inclusion of HPbI_3_ or dimethylammonium iodide (DMAI) additives regulates the crystallization rate and stabilizes the photoactive black phases of the CsPbI_3_ perovskite polymorphs β (tetragonal) and γ (orthorhombic). Moreover, other techniques, including metal cation doping,^[^ ^47^, ^48^ ^]^ surface treatments,^[^ ^49^, ^50^, ^51^ ^]^ molecular additives,^[^ ^52^, ^53^, ^54^, ^55^, ^56^ ^]^ and interfacial passivation approaches,^[^ ^57^, ^58^, ^59^, ^60^, ^61^ ^]^ can effectively stabilize the black phases and enable impressive performances under ambient conditions and low temperatures. Although the methods hold promise, a noticeable discrepancy exists between the theoretical potential and practical implementation of inorganic perovskite.

This comprehensive review explores recent developments, ongoing challenges, and future potential for the low‐cost fabrication of highly efficient, stable, and scalable inorganic PSCs. While several published reviews on inorganic perovskites exist, none specifically address the potential for low‐cost fabrication.^[^ ^18^, ^21^, ^23^, ^39^, ^55^ ^]^ By this, we refer to the ability to fabricate inorganic PSCs under ambient conditions using low‐temperature processing steps, representing the most cost‐effective commercialization manufacturing pathway. Beginning with a detailed overview of the material background of inorganic PSCs, this review examines progress in fabrication under ambient conditions, emphasizing the influence of factors such as moisture, oxygen, and temperature on perovskite film formation and quality. Understanding and controlling these factors is critical for achieving high efficiency, stability, and scalability in fabricating inorganic PSCs. The review further provides the latest updates on state‐of‐the‐art high‐efficiency inorganic PSCs, advancements in upscaling to large‐area devices, and improvements in device stability. We conclude by offering key insights into the challenges and opportunities in inorganic PSC research as it progresses toward commercialization.

We acknowledge that concerns regarding lead toxicity and its safety implications for the environment and health have driven increased research into novel inorganic PSCs. While certain lead‐free inorganic perovskites—or perovskite‐inspired materials—such as CsSnI_3_, Cs_3_Bi_2_I_9_, Ag_3_BiI_6_, and Cs_2_AgBiBr_6_ exhibit impressive potential,^[^ ^62^, ^63^, ^64^, ^65^ ^]^ we have chosen not to include these materials in this review. The decision stems from the fact that they remain in the early stages of development, with efficiencies still far behind those of lead‐halide inorganic PSCs. Accordingly, this review delves exclusively into CsPbX_3_ perovskites, which we also refer to as inorganic PSCs henceforth.

## 2 Material Background

The exploration of inorganic halide perovskites began in the 1890s, significantly before Weber discovered organic–inorganic hybrid Pb‐halide perovskites in 1978.^[^ ^66^ ^]^ In 1893, Wells was the first to create Pb‐halide compounds such as CsPbX_3_ or RbPbX_3_ (X = Cl, Br, I) from solutions containing Pb halides and Cs or Rb.^[^ ^67^ ^]^ In 1957, Møller discovered the perovskite structure of CsPbCl₃ and CsPbBr₃, with a propensity to transform from distorted tetragonal into a pure cubic form at high temperatures.^[^ ^68^, ^69^ ^]^

### 2.1 Crystal Structure

PSCs have the general formula AMX_3_ with well‐defined crystal structures. Within the structures, a metallic cation M is located at the center of the octahedra with a coordination number of 6, enveloped by an octahedral [MX_6_]^4−^ cluster comprising X^−^ anions at the face centers. A cation occupies the eight corners of the cube with a coordination number of 12. The stability of the perovskite structure depends on the Goldschmidt tolerance factor,^[^ ^42^, ^43^ ^]^ which measures the fit of different ions in the crystal structure of the material, assuming that all ions are rigid and closely packed spheres. A perfectly cubic perovskite phase requires a tolerance factor of *t* = 1. A tolerance value between 0.8 and 1 can still form a perovskite phase with a slight distortion of [BX_6_]^4−^ octahedra. When the factor is between 0.9 and 1.0, materials adopt a cubic structure, while either orthorhombic or tetragonal phases dominate at lower (0.7 < *t* < 0.9) or higher (*t* > 1.0) values, respectively.

**Figure 2:**
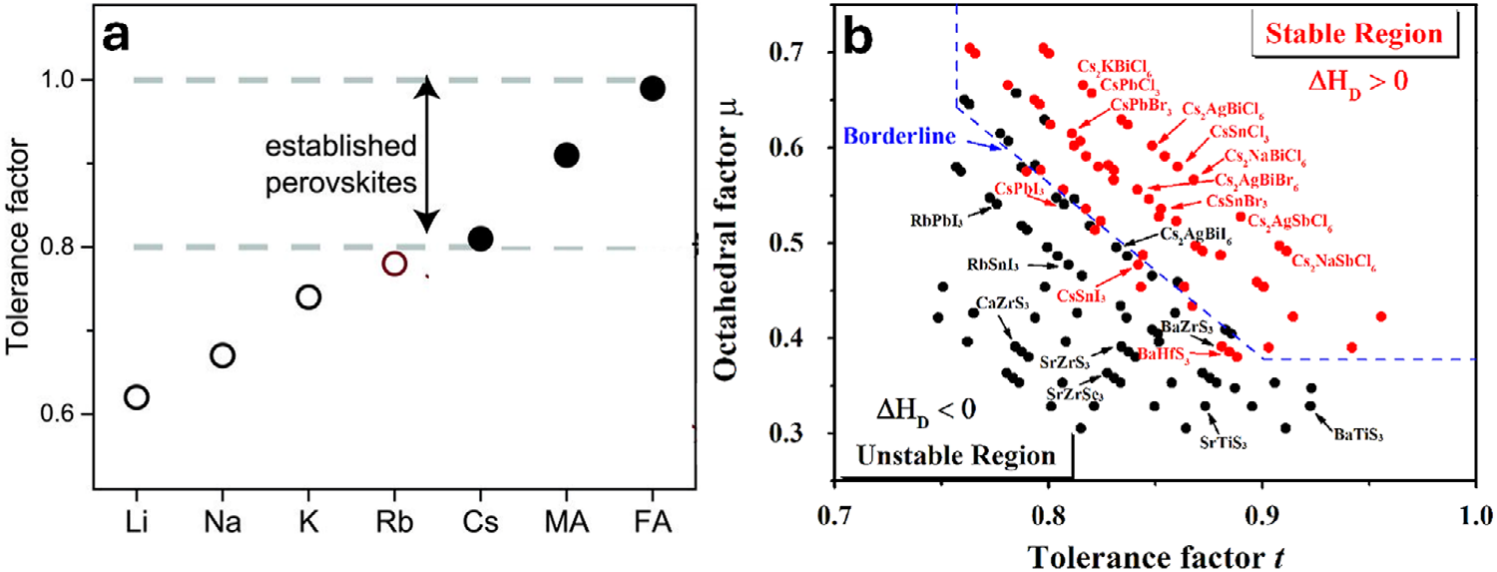
a) Tolerance factor analysis with respect to the types of organic and inorganic cations that form APbI_3_ perovskite. Adapted with permission.^[^ ^70^ ^]^ Copyright 2016, American Association for the Advancement of Science. b) Simulation of 138 inorganic perovskite compounds, with stable and unstable compounds indicated by red and black dots on a (*t*, *µ*) map where decomposition energies are *δH*_D_ > 0 and *δH*_D_ < 0, respectively. Adapted with permission.^[^ ^71^ ^]^ Copyright 2017, American Chemical Society.

Accordingly, perovskite materials exhibit different crystal structures depending on their formation temperatures and tolerance factors (**Figure** **2**a). For CsPbI_3_, a symmetric cubic‐phase perovskite with a tolerance factor between 0.9 and 1.0 can form at high temperatures. However, less symmetric tetragonal or orthorhombic phases are preferably formed at low temperatures with a lower tolerance factor. For instance, CsPbI₃, with a tolerance factor of ≈0.8, displays a yellow orthorhombic phase at room temperature due to lattice distortion. Understanding the relationship between the tolerance factor (*t*), crystal structure, octahedral factor (*µ*), and temperature is crucial for designing and optimizing perovskite materials for various applications. Recent studies have suggested that correlating these factors can be a reliable method for predicting the stability of perovskite structures. Sun and Yin illustrated this by simulating a map of (*t*, *µ*) for 138 perovskite compounds (Figure 2b), concluding that perovskites are stable within the ranges of 0.813 < *t* < 1.107 and 0.377 < *µ* < 0.895. The following expressions define the parameters *t* and *µ*

(1) $$t=\frac{R_{A}+R_{X}}{\surd 2\left(R_{M}+R_{X}\right)}$$

(2) $$\mu =\frac{R_{B}}{R_{X}}$$

where *R*_A_, *R*_M_, and *R*_X_ are the radii of the corresponding ions in the AMX_3_ structure.

From Figure 2b, it can be noted that the instability of black CsPbI_3_ (a well‐explored inorganic Pb‐halide perovskite) is attributed to its borderline *t* value of 0.81, which is just above the permissive range, mainly due to the smaller Cs^+^ cation that has difficulty maintaining the [PbX_6_]^4−^ octahedra, leading to considerable lattice distortion causing structural instability. Various studies have been conducted to determine the stability of both α‐ and δ‐CsPbI_3_ polymorphs in relation to their binary halides, CsI and PbI_2_.^[^ ^72^, ^73^, ^74^ ^]^ The formation enthalpies of the two polymorphs have been calculated, and both are stable as binary halides. At room temperature, the formation enthalpy of δ‐CsPbI_3_ is more negative (−16.93 ± 0.87 kJ mol^−1^) than that of α‐CsPbI_3_ (−2.83 ± 0.90 kJ mol^−1^), indicating that α‐CsPbI_3_ is metastable at a lower temperature.^[^ ^75^ ^]^ As shown in Figure 2, the findings provide essential insights into the stability of CsPbI_3_ polymorphs, which is crucial for their potential applications in various fields, such as solar cells and optoelectronic devices. Owing to their smaller radii, other inorganic cations, including Rb^+^ or alkali metal cation Pb‐halide compounds, do not attain the Goldsmith tolerance factor to form cubic‐phase perovskites.

**Figure 3:**
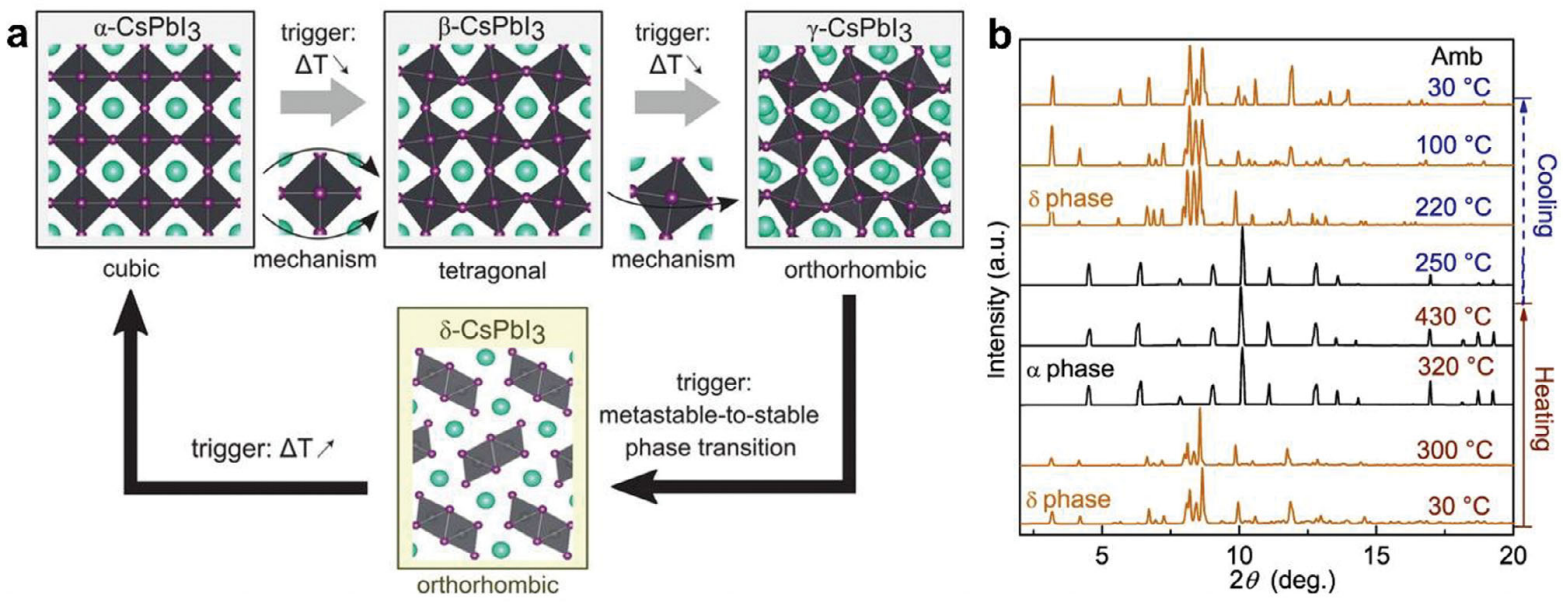
a) Crystal structure of different phases and their relative phase transitions. Transitions between the black phases are governed by the local Pb‐centered octahedral (black) distortions, depicted using one Pb atom at the center and six I atoms at the edges (purple), confining the Cs cations (cyan). Adapted with permission.^[^ ^76^ ^]^ Copyright 2019, American Association for the Advancement of Science. b) X‐ray diffraction (XRD) patterns of CsPbI_3_ collected through both heating and cooling cycles at ambient pressure. The intensity observed between α‐ and γ‐CsPbI_3_ XRD patterns varies due to grain size and orientation differences, which are influenced by grain growth and texture formation at elevated temperatures. Reproduced under the terms of the CC‐BY license.^[^ ^77^ ^]^ Copyright 2021, Springer Nature.

Recognizing that the stability of inorganic perovskites, particularly the CsPbI_3_ black phase, processed at high temperatures, does not make them reliable replacements for hybrid perovskites is essential. The main challenge lies in finding a solution to stabilize the black phase, which is photoactive under ambient conditions and at room temperature. CsPbI_3_ undergoes various crystalline transformations at different temperatures (**Figure** **3**a). At room temperature, CsPbI_3_ adopts a nonphotoactive orthorhombic form that appears yellow and is commonly referred to as the δ‐phase. With increasing temperature, CsPbI_3_ changes its crystal structure and transforms into black orthorhombic γ‐phase, tetragonal β‐phase, and cubic α‐phase over 175, 260, and 360 °C, respectively (Figure 3b). After cooling to room temperature, CsPbI_3_ returns to its original nonphotoactive yellow δ‐phase, one of the main reasons being that the Cs element is very small to maintain the PbI_6_ polyhedra in cubic α‐CsPbI₃ at average room temperature. Thus, CsPbI₃ quickly deteriorates and transforms into orthorhombic δ‐CsPbI₃ when cooled.

### 2.2 Optoelectronic Properties

An absorber material should satisfy specific criteria to achieve high‐efficiency solar cells. The criteria include an optimal bandgap, high absorption coefficient, long carrier diffusion lengths, excellent charge‐transport capabilities through electron and hole mobilities, and high defect tolerance.

#### 2.2.1 Electronic Structure

**Figure 4:**
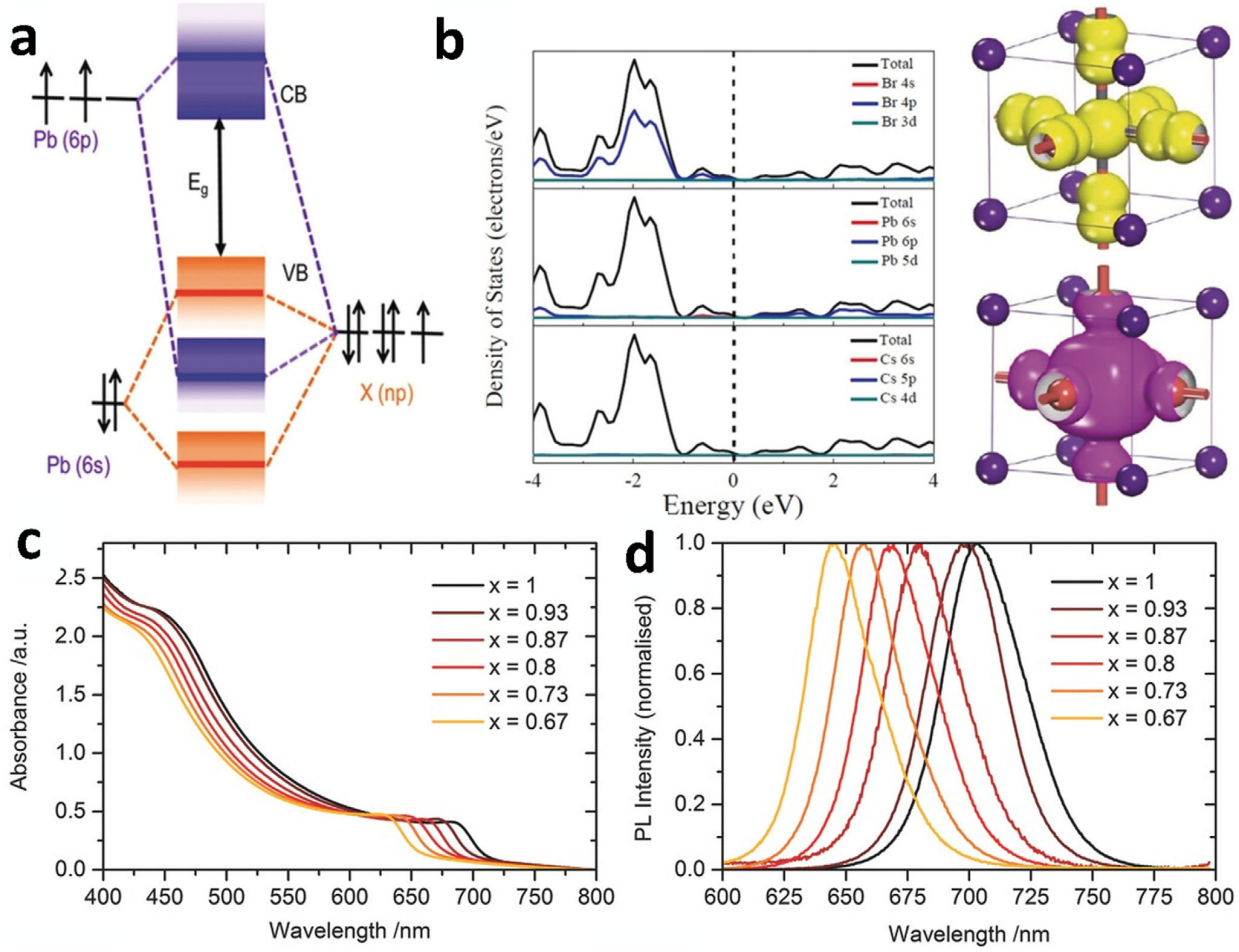
a) VB and CB are formed through bonding and antibonding orbitals of APbX_3_. Reproduced with permission.^[^ ^81^ ^]^ Copyright 2016, American Chemical Society. b) Density of states for cubic CsPbBr_3_, highlighting the contributions of each element to the energy bands. Additionally, electronic profiles for VBM and CBM are included. Reproduced with permission.^[^ ^29^ ^]^ Copyright 2016, John Wiley and Sons. c) Optical absorption and d) photoluminescence (PL) spectra of CsPbI*_x_* Br_3–_ *_x_* with altering concentrations of I “*x*.” Reproduced with permission.^[^ ^87^ ^]^ Copyright 2016, John Wiley and Sons.

Based on density functional theory (DFT) calculations for CsPbX_3_ perovskites,^[^ ^78^, ^79^, ^80^ ^]^ valence band maximum (VBM) primarily comprises antibonding hybridized Pb 6s and X np orbitals, with X np being the most prominent contributor (**Figure** **4**a). Conversely, the conduction band minimum (CBM) of CsPbX_3_ comprises antibonding mixing of the Pb 6p and X np orbitals, with Pb 6p having the most significant contribution,^[^ ^81^ ^]^ including a direct bandgap with a p‐to‐p transition, leading to a high absorption coefficient that makes CsPbX_3_‐type perovskites highly versatile for optoelectronic applications. Additionally, Pb cations in the conduction band play a significant role in perovskite semiconductors, whereas elements with lone pair electrons contribute to p properties in the conduction band.

Studies have demonstrated that halides do not significantly impact the nature of the band structure (direct or indirect band transition) of CsPbX_3_ perovskites, except for affecting bandgap values, which are influenced by relativistic correction and spin–orbit interaction.^[^ ^82^ ^]^ Moving from I (5p) to Br (4p) to Cl (3p), the energies of the halide np orbitals decrease, leading to a shift of VBM toward more positive potentials.^[^ ^81^ ^]^ Comparatively, CBM shifts slightly toward higher energy levels, widening the bandgap. Furthermore, Cs^+^ has minimal direct influence on the electronic structure of the band edge, resembling the role of organic A cations in hybrid perovskites.^[^ ^29^, ^82^ ^]^ Thus, the transition and recombination of electrons and excitons are limited within PbX_6_^4‐^ octahedra, implying minimal effects on electronic structure with structural tilting or phase change (Figure 4b). Nevertheless, CsPbI₃ perovskites display noticeable structural and electronic changes across different phases due to the larger ionic radii of I, while CsPbBr₃ and CsPbCl₃ exhibit minimal variations in their structures.^[^ ^83^, ^84^ ^]^ Furthermore, band dispersion can predict other electronic properties, including the effective masses of electrons and holes and excitonic binding energies, which impact charge carrier mobility and separation.^[^ ^85^, ^86^ ^]^

The transformation of the photoactive cubic phase to the orthorhombic yellow phase has a distinct impact on band structure, resulting in a broader bandgap and lower carrier mobilities.^[^ ^88^ ^]^ Through additive synthetic strategies and high‐pressure‐assisted processing, researchers have maintained the black phase by tilting the octahedra and controlling the electronic properties.^[^ ^89^, ^90^, ^91^ ^]^ However, gaining a comprehensive understanding of the optoelectronic properties of perovskite materials at the atomistic level requires further emphasis on theoretical and experimental studies of their electronic structure.

#### 2.2.2 Optical Absorption and Bandgap

Photoabsorber materials in solar cells should exhibit outstanding photon absorption properties to achieve high PCEs. Similar to hybrid perovskite materials, inorganic perovskite materials demonstrate distinct (over 10^4^ cm^−1^) absorption coefficients onsets beyond bandgaps (Figure 4c).^[^ ^92^ ^]^ Considering the thickness (≈500–600 nm) and absorption coefficient of inorganic perovskite active layers in highly efficient devices, the devices maintain a sharp quantum efficiency with thinner photoactive layers compared to Si solar cells. As a vital benefit of the method, the minimum diffusion length necessary to gather all carriers is established, which is ideally multiple times the film thickness. Consequently, using extremely thin films corresponds to a reduced carrier diffusion length.^[^ ^93^, ^94^ ^]^ However, data on the absorption coefficient and carrier mobility of inorganic Pb‐halide perovskites are relatively limited.

Furthermore, synthetic conditions such as the annealing environment, temperature, and duration significantly influence the optical properties of CsPbX_3_.^[^ ^95^ ^]^ Contrary to hybrid perovskites, an excess of precursors (CsI or PbX_2_) exhibits a detrimental impact on optical absorption. Thus, a stoichiometrically balanced (1:1) CsPbX_3_ offers a much higher absorption coefficient and broader absorption.^[^ ^96^ ^]^

The optical absorption properties of a material are primarily determined by its bandgap. CsPbIBr_2_ and CsPbBr_3_ have wider bandgaps, ≈2.05 and 2.3 eV, respectively, in comparison to CsPbI_3_, which has a relatively narrower bandgap of ≈1.73 eV in its cubic phase (α‐CsPbI_3_).^[^ ^15^, ^87^, ^97^, ^98^, ^99^ ^]^ Further, indium‐doped CsPbI_3_ presents improved absorbance in the 400–700 nm range, making it a promising choice in optoelectronic devices.^[^ ^100^, ^101^ ^]^ Furthermore, Singh et al. used CsPbX_3_ inorganic perovskites for light‐harvesting purposes owing to their adaptable bandgap covering the visible spectrum.^[^ ^102^ ^]^ Researchers have studied the refractive index, extinction coefficient, and dielectric function at various angles of incidence in the visible spectrum. At 435 nm, the refractive index of CsPbI_3_ was recorded as 2.46, suggesting minimal light loss caused by reflection at the front of the active layer. Consequently, Cs‐based Pb‐halide solar cells can serve as effective antireflection coatings for tandem solar cells. Finally, X‐ray diffraction (XRD) analysis of the cubic‐phase *Pm‐3m* microstructure of CsPbI_3_ revealed a sharp absorption edge and photoluminescence emission close to the infrared region, with *E*_g_ = 1.67 eV.

Optimizing the optical and electronic characteristics of mixed‐halide perovskites CsPbI*_x_* Br_3−_ *_x_* with different I/Br ratios is another approach to improving light absorption and enhancing CsPbX_3_ stability.^[^ ^87^ ^]^ Sutton et al. observed a linear relationship between I content “*x*” and absorption onset in CsPbI*_x_* Br_3−_ *_x_*, following Vegard's law—the development of solid solutions of I–Br mixtures (Figure 4c,d).^[^ ^87^ ^]^

#### 2.2.3 Carrier Mobilities and Lifetime

Selective absorber materials with high mobilities, long carrier lifetimes, and optimal diffusion lengths are critical for achieving the highest PV performance. The properties enable efficient charge extraction and prevent trap‐assisted recombination. Limited studies on the mobilities and lifetimes of CsPbX_3_ are available in the literature; however, studies suggest that inorganic perovskite films have mobilities comparable to those of their organic–inorganic hybrid counterparts.^[^ ^103^, ^104^, ^105^ ^]^ Zhu et al. conducted a comprehensive study on the properties of carriers at the band edges of APbBr_3_ macrocrystals with various A cations, including MA^+^, FA^+^, and Cs^+^. The study revealed that the type of A‐cation had a negligible impact on carrier transport.^[^ ^103^ ^]^ However, the halide component significantly affected carrier mobility. For example, the electron/hole mobility of spin‐coated polycrystalline CsPbI_3_ films measured with a time‐resolved microwave conductivity (TRMC) technique is reported as ≈25 cm^2^ V^−1^ s^−1^.^[^ ^106^, ^107^ ^]^ A slightly lower value of 10 cm^2^ V^−1^ s^−1^ is reported for spin‐coated CsPbI_2_Br films.^[^ ^108^ ^]^ Conversely, CsPbBr_3_ quasi‐monocrystalline films fabricated via the four‐step hot pressing and slow cooling method have demonstrated values of 38 cm^2^ V^−1^ s^−1^, larger than the fast‐cooled control samples (12 cm^2^ V^−1^ s^−1^) measured using the combination of the space‐charge limited current (SCLC) method.^[^ ^105^ ^]^

Among different compositions of inorganic perovskites, a significant variation in mobility can occur depending on the nature of the films, such as the grain size, single crystal versus multicrystalline structure, and chemical and stoichiometric composition of inorganic perovskites. Additionally, different measurement methods can cause variations in mobility values. In the case of CsPbBr_3_ single crystals, larger crystals exhibit exceptional charge–carrier mobilities. Notably, the time‐of‐flight technique calculates a high electron mobility of 2290 cm^2^ V^−1^ s^−1^, while the SCLC method sandwiching CsPbBr_3_ single crystals between Au electrodes yields electron and hole mobilities of 2240 and 2060 cm^2^ V^−1^ s^−1^, respectively.^[^ ^109^ ^]^ However, Hall effect measurements resulted in CsPbBr_3_ single crystals having a significantly lower hole mobility of 143 cm^2^ V^−1^ s^−1^ but electron mobility of 2270 cm^2^ V^−1^ s^−1^, close to the value obtained by the SCLC method.^[^ ^54^, ^59^, ^103^, ^104^ ^]^ Ordered CsPbIBr_2_ single crystals grown from solution exhibit a high carrier mobility of 2574 cm^2^ V^−1^ s^−1^ but display remarkable anisotropy when measured with the SCLC technique.^[^ ^60^, ^105^, ^110^ ^]^

Similar to charge‐carrier mobilities, the carrier lifetime of CsPbX_3_ can vary owing to the differences in perovskite processing and measurement methods. Hutter et al. compared evaporated and spin‐coated CsPbI_3_ and reported that the evaporated films had an approximate carrier lifetime of 10 µs while the spin‐coated ones had ≈0.2 µs, based on TRMC measurements.^[^ ^106^ ^]^ Simultaneously, pump‐probe time‐resolved terahertz spectroscopic analysis of solution‐processed polycrystalline^[^ ^106^, ^107^ ^]^ CsPbI_3_ perovskite films exhibit monomolecular decay‐dominated lifetimes of over 20 ns.^[^ ^107^ ^]^ In contrast, the photoconductivity measurements of solution‐grown single crystals of CsPbBr_3_ show carrier lifetimes of ≈2.5 µs (for single crystals)^[^ ^61^ ^]^ and ≈44 ns for spin‐coated top‐modified CsPbBr_3_ films,^[^ ^62^ ^]^ and 14 ns for CsPbI_2_Br films.^[^ ^111^ ^]^

Dirin et al. studied the mobility–lifetime products of CsPbBr_3_ single crystals, revealing that CsPbBr_3_ exhibits a relatively lower mobility–lifetime product (≈2 × 10^−4^ cm^2^ V^−1^) than hybrid perovskites (1–1.8 × 10^−2^ cm^2^ V^−1^).^[^ ^65^, ^66^, ^67^ ^]^ The shorter carrier lifetime of CsPbBr_3_ compared to hybrid perovskites (up to 500 µs) is attributed to this difference, as the formation of asymmetric electric fields by asymmetric organic cations aids in carrier separation, leading to increased carrier lifetimes in the case of hybrid perovskites.^[^ ^66^, ^68^, ^69^, ^70^, ^112^ ^]^ The mobility–lifetime product is crucial for efficiently extracting charge carriers prior to recombination. Therefore, a lower carrier lifetime (tens of nanoseconds) of Br‐rich polycrystalline films in inorganic Pb perovskites can be considered a major constraint for their PV performance as the surface trap states increase.

Regarding carrier diffusion length, Dastidar et al. analyzed transient absorption decay and estimated the diffusion length of solution‐processed CsPbI_3_ as 1 µm, assuming balanced electron and hole transport.^[^ ^56^ ^]^ Contrarily, the diffusion lengths of electrons and holes in single‐crystal CsPbBr_3_ were ≈10 and 12 µm, respectively.^[^ ^109^ ^]^ Nanocrystals of CsPbBr_3_ exhibited both electron and hole diffusion lengths of over 9.2 µm when measured using terahertz conductivity.^[^ ^71^, ^113^ ^]^ Accordingly, monocrystalline CsPbBr_3_ films showcase a substantial carrier diffusion length of ≈4.2 and 5.9 µm for hole and electron diffusion length, respectively.^[^ ^114^ ^]^ Despite these promising findings, electron beam‐induced current measurements of polycrystalline CsPbBr_3_ films indicate a significantly shorter electron diffusion length of 80 nm.^[^ ^115^ ^]^ Owing to their lower trap densities, single crystals and quantum dots (QDs) generally exhibit greater mobility, longer charge lifetimes, and longer diffusion lengths than polycrystalline films.

#### 2.2.4 Defects

Although a photoactive material has a desirable bandgap, optical absorption, and carrier mobility, defects are critical for determining charge separation and transport. Perovskite film defects create trap levels that impede charge carrier flow in PSCs. Minimizing the defect density or reducing carrier trapping by defects is crucial for ensuring the longevity of the carrier materials. Thus, understanding the nature of defects is vital to ensuring optimal photovoltaic performance of materials. CsPbX_3_ crystals possess three distinct types of point defects. In the lattice structures, three intrinsic point defects exist: vacancies (V_Cs_, V_Pb_, and V_I_), interstitials (Cs_i_, Pb_i_, and I_i_), and antisite substitutions (Cs_Pb_, Cs_I_, Pb_Cs_, Pb_I_, I_Cs_, and I_Pb_), where atoms occupy either the in‐between space or the wrong site in a lattice. Notation A_B_ indicates that A has been replaced by B.^[^ ^116^, ^117^ ^]^

Two types of point defects have been identified based on their position within a bandgap. Shallow‐level states such as V_Cs_, V_I_, V_Pb_, Cs_i_, I_i_, and Cs_Pb_ require lower activation energies. Conversely, deep‐level states such as Pb_I_, Pb_i_, I_Pb_, and I_Cs_ are closer to the center of the bandgap than CBM or VBM, making detrapping of charge carriers difficult once captured in deep defect states. Although shallow traps do not present significant electronic obstacles, they are problematic when converted into deep defects that serve as scattering and recombination centers. For example, when V_I_ captures an electron and becomes negatively charged, the nearby Pb atoms bond to create a new deep trap level, which can significantly affect the transport of free carriers in CsPbX_3_ perovskites.

I‐rich inorganic perovskite materials have charge carriers that persist for extended periods, indicating that perovskite imperfections are less detrimental to their electrical properties than traditional semiconductors. The high tolerance of inorganic perovskites to defects can be attributed to three factors. First, the majority of natural point defects are minor, with only a few being severe. Second, the flexibility of inorganic lattice enables electron and hole separation in the presence of defects, restricting interactions to low‐frequency motions. Third, given the high dielectric constant of perovskite materials, indicating the ability to neutralize electrostatic disturbances, adequate protection against the disruptive effects of charged defects is provided.

Perovskite film defects can impede the flow of charge carriers in perovskite devices by creating trap levels. The main source of energy loss in devices involves defect‐assisted Shockley–Read–Hall nonradiative recombination, which captures photogenerated carriers and reduces the diffusion length of free charge carriers, ultimately decreasing device efficiency. Additionally, point defects affect the Fermi level of the photoabsorber layer in PSCs. If the Fermi energy is pinned at a position where the compensation defect formation energy is zero, both *V*_oc_ and PCE are low. Therefore, minimizing or suppressing the nonradiative recombination channels caused by defects is crucial to enhancing the efficiency of PSCs. In DFT calculations by Kang and Wang, deep‐level defects in inorganic perovskite materials continued to exhibit significantly elevated formation energies.^[^ ^118^ ^]^ The result was attributed to the deficiency of bonding–antibonding interactions between the valence and conduction bands in the defects, which was deemed essential for enhanced defect tolerance in inorganic perovskites.

## 3 Device Architectures

**Figure 5:**
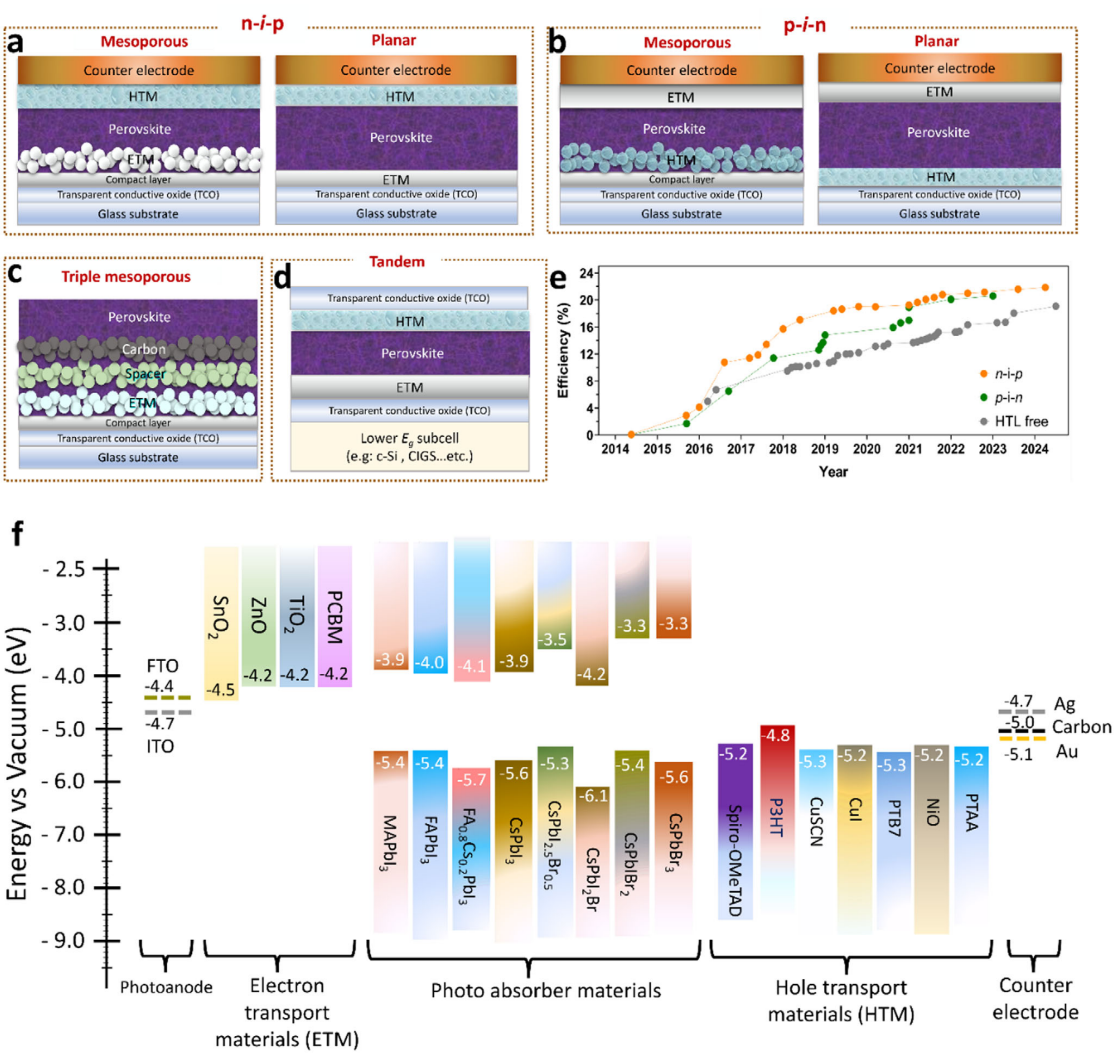
PSC architectures: a) n–i–p, b) p–i–n, c) triple mesoporous HTL‐free, d) tandem, e) evolution of efficiency from each architecture over the years, and f) energy levels of MAPbI_3_, FAPbI_3_, selected inorganic perovskites, perovskite‐like materials, electron‐ and hole‐transport materials, and the work functions of the commonly used electrode materials.

Inorganic PSCs feature a layered stack architecture comprising a sub‐micrometer‐thick perovskite material as an intrinsic light‐absorbing layer. The layer is surrounded by two charge‐carrier layers, p‐type (hole‐transport layer, HTL) and n‐type (electron transport layer, ETL), on either side of the light‐absorbing layer. The “p‐type” and “n‐type” layers transport holes and electrons, respectively. The layers are sandwiched between two charge‐collector electrodes. The sequence of the charge‐transport layer to the substrate defines two commonly used architectures: n–i–p and p–i–n. Both n–i–p and p–i–n types can be formed in planar or mesoporous structures (**Figure** **5**). In a planar architecture, both the ETL and HTL layers are nominally flat (Figure 5a). Contrarily, a mesoporous structure is derived from dye‐sensitized solar cells and uses a mesoporous scaffold of a certain ETL or HTLs before depositing a perovskite absorber to increase the interface between the two layers.

In the planar n–i–p architecture, ETLs generally comprise metal oxide semiconductors such as TiO_2_ and SnO_2_, which are directly deposited on transparent conductive oxide (TCO)‐coated glass, mainly In‐doped SnO_2_ (ITO) or F‐doped SnO_2_ (FTO). The oxides efficiently collect and transport electrons while blocking holes. In the mesoporous n–i–p structure, a mesoporous metal oxide semiconductor ETL is used in addition to a compact planar ETL, which enhances the surface area between the perovskite absorber and ETL and promotes electron transport. Typically, TiO_2_ forms a mesoporous scaffold, but other oxides have been reported recently, such as ZrO_2_ or Al_2_O_3_.^[^ ^119^ ^]^ HTL is positioned above the inorganic perovskite layer and transports holes while blocking electrons. Typically, the thicknesses of a mesoporous ETL and inorganic perovskite layers are maintained at ≈500 nm, largely defined by the thickness of the mesoporous ETL layer. However, the high preparation temperature (>400 °C) of the mesoporous layer limits the use of FTO as a transparent conductive layer, increases production cost, and makes the process unsuitable for low‐cost fabrication of flexible substrates, such as polyethylene terephthalate (PET). Typically, 2,2′,7,7′‐tetrakis[*N*,*N*‐di (4‐methoxyphenyl)amino]‐9,9′‐spirobifluorene (spiro‐OMeTAD), a conventional HTL material, is used to extract holes and transport them to metallic or carbon counter electrodes.^[^ ^4^ ^]^

In the p–i–n or inverted architecture, HTL is deposited over the TCO comprising materials such as nickel oxide (NiO*_x_*), poly(3,4‐ethylene‐dioxythiophene) polystyrene sulfonate (PEDOT:PSS), or poly[bis(4‐phenyl)(2,4,6‐trimethylphenyl)amine (PTAA), followed by the deposition of a perovskite light absorber layer (Figure 5b). Further, ETL such as phenyl‐C61‐butyric acid methyl ester (PCBM) is deposited over the perovskite layer, and the device is completed by depositing a metallic or carbon counter electrode.^[^ ^120^, ^121^ ^]^ Furthermore, a triple mesoporous HTL‐free structure is introduced when the design is extended. The perovskite absorber of the structure is impregnated into a triple‐layer mesoporous scaffold consisting of n‐type material in contact with a glass|TCO electrode, an insulator spacer (Al_2_O_3_), and C as the hole collector and counter electrode (Figure 5c). In addition to the low fabrication complexity of the planar configuration, the structure is widely integrated with first‐ or second‐generation solar cells to produce tandem solar cells (Figure 5d).

The prevalent type of PSCs utilizes a glass or TCO substrate to support an n‐type contact, whereas inverted p–i–n devices have a reversed arrangement of components (Figure 5a,b). Optimizing the deposition methods for all components is crucial to achieving optimal performance for both architectures. Electron transport materials, such as fullerene (C60) and [6,6]‐phenyl‐C_61_‐butyric acid methyl ester (PC_60_BM), are commonly used in p–i–n devices. The energy levels of such materials align with inorganic perovskite composition and other functional materials used in hybrid PSCs, such as MAPbI_3_ and FAPbI_3_ (Figure 5f). Such materials are crucial for transporting electrons from the perovskite layer to the n‐type contact, enabling light conversion into electrical energy. Conversely, several hole transporters are preferred for high‐performance PSCs, including poly[3‐(4‐carboxybutyl)thiophene‐2,5‐diyl] (P3CT‐N), spiro‐OMeTAD, poly(3‐hexylthiophene) (P3HT), PTAA, NiO*_x_*, CuI, and CuSCN. Hole transporters complete the circuit by transporting holes from the perovskite layer to the p‐type contact, enabling current flow. Therefore, optimizing electrons and HTL is critical for achieving high‐performance PSCs. Ongoing research focuses on developing novel and improved materials and deposition techniques that instill hope for enhancing the efficiency and stability of such devices.

## 4 Fabrication of Inorganic Perovskite Films

To achieve highly efficient PSC devices, fabricating uniform and pinhole‐free perovskite layers with high crystallinity, large grains, and uniform and dense surface morphologies is crucial. The characteristics of perovskite films are strongly influenced by the fabrication method. Several promising fabrication techniques, such as spin coating,^[^ ^122^, ^123^ ^]^ hot‐air‐assisted deposition,^[^ ^124^, ^125^ ^]^ spray‐assisted deposition,^[^ ^126^, ^127^ ^]^ slot die coating,^[^ ^128^ ^]^ and vacuum‐based thermal evaporation^[^ ^129^, ^130^ ^]^ have been used for the deposition of inorganic perovskite absorber films.

Solution‐based fabrication of PSCs involves solvent engineering techniques that produce high‐quality perovskite films and high‐performance PSC devices. The cost‐effective approach is suitable for high‐throughput vacuum‐free scale‐ups using batch‐to‐batch or continuous roll‐to‐roll production. In a laboratory, the solution‐based fabrication method is executed using a spin‐coater in a single or two‐step process to fabricate an inorganic perovskite layer, depending on the solution processability of individual precursors.

The one‐step deposition method involves depositing a perovskite precursor solution onto a substrate, followed by annealing to form a perovskite film. To create a perovskite film, the precursors are dissolved in a solvent (typically DMF) and applied to the substrate using a spin‐coater, spinning at a specific rotation rate for a set period. The process generates an intermediate adduct treated with antisolvents or a gas flow to extract the polar solvents, achieving supersaturation and inducing nucleation, followed by thermal annealing to produce the final perovskite film. The two‐step deposition method involves depositing a Pb‐based precursor solution (PbI_2_ or PbAc_2_) onto the substrate, followed by annealing to form a PbX_2_ film. In the second step, a solution of the second precursor material (such as MAI, FAI, CSI, and CSBr) is deposited onto the predeposited Pb‐based precursor film, followed by annealing, converting the PbX_2_ into perovskite film. In the two‐step process, the morphology and crystallinity of the PbX_2_ films play significant roles in the crystallization of the perovskites. The annealing temperature required to achieve α‐phase CsPbI_3_ is typically 310 °C, while mixed‐halide formulations require a lower annealing temperature of ≈250 °C.

Current state‐of‐the‐art hybrid halide perovskites are based on formamidium‐cesium double‐cation perovskites. In the formulation, the addition of CsI plays a crucial role in suppressing the crystallization of PbI_2_ by forming δ‐CsPbI_3_, which reacts with FA halides, accelerating the crystal growth via Ostwald ripening and forming films with large grains and minimal pinholes.^[^ ^131^, ^132^ ^]^ The strategy has led researchers to develop new record efficiency because the thermal instability of the black FAPbI_3_ phase with its upper borderline tolerance factor reduces with the incorporation (<0.2) of smaller‐sized Cs^+^ and a small amount (<0.1) of Rb^+^. However, a higher amount of Cs^+^ or Rb^+^ generates phase segregation, forming nonphotoactive phases of CsPbI_3_ or RbPbI_3_ due to a significant lattice mismatch.^[^ ^70^ ^]^ Contrarily, a similar approach utilizing larger cations to boost the tolerance factor and enhance the stability of the black CsPbI_3_ phase yields unfavorable results when incorporating FA^+^.^[^ ^133^ ^]^ However, this approach has proven effective with the use of dimethylammonium cations (DMA^+^), which results in the formation of Cs_1−_ *_x_* DMA*_x_* PbI_3_ and reduces the processing temperature of CsPbI_3_.^[^ ^134^, ^135^ ^]^ Simultaneously, the Cs_4_PbI_6_‐mediated synthesis of the FA_0.15_Cs_0.85_PbI_3_ perovskite improves tolerance factor and stability.^[^ ^136^ ^]^

The solubility of precursors and crystallization dynamics differ significantly for CsPbX_3_, and utilizing different halide compositions requires careful solvent engineering. For instance, CsPbI_3_ dissolves in DMF, while CsPbI_2_Br requires higher‐boiling‐point solvents such as DMSO for one‐step fabrication. Due to the limited solubility of Cs and Pb halides (specifically Br‐rich) in commonly used solvents such as DMF and DMSO, researchers have explored coordination compounds formed by the interplay between the Lewis base (DMSO or DMF) and the Lewis acid PbI_2_.^[^ ^137^, ^138^, ^139^, ^140^ ^]^ The adduct formed in DMSO is known to be more stable than that formed in DMF because of the stronger Lewis basicity generated by the electron‐donating methyl groups in DMSO. By tailoring interactions with solvents such as MAAc,^[^ ^141^, ^142^ ^]^ *N*‐methyl‐2‐pyrrolidone (NMP),^[^ ^143^ ^]^ acetonitrile, and 2‐methoxy ethanol mixtures,^[^ ^159^ ^]^ researchers have regulated perovskite crystallization to improve film quality and performance.

Unlike CsPbI_3_, which can be deposited in a single one‐step process, Br‐rich compositions such as CsPbIBr_2_ and CsPbBr_3_ necessitate a two‐step or sequential deposition owing to solubility and coordination issues of precursor components such as CsBr and PbBr_2_. The low solubility of CsBr and uncontrolled crystallization cause unwanted secondary phases, such as Pb‐rich (CsPb_2_Br_5_) and Cs‐rich (Cs_4_PbBr_6_) phases. Sequential deposition circumvents the challenge of solubility and coordination, enabling the formation of a more homogeneous and stoichiometric perovskite film, leading to improved device performance.

Selecting a deposition method for perovskite films is crucial for achieving high‐quality films and high‐performance devices. Additionally, the fabrication environment significantly influences device performance and stability. Currently, spin‐coating perovskite films in an inert environment are ubiquitously used to fabricate laboratory devices using various crystallization control strategies. However, spin coating is not scalable, and the films achieved are often not translatable in a scalable fabrication method due to the different nature of film formations. Furthermore, using an inert environment is a technical and commercial obstacle to employing high‐throughput, large‐scale, low‐cost manufacturing, and harnessing the solution‐processability of the perovskite precursors using industrial printing and coating methods.

While spin coating on a discrete substrate in an inert environment using high‐temperature steps (often above 310 °C) and prolonged annealing conditions (exceeding 1 h) results in record efficiencies for inorganic PSCs, translating these processes into an industrial‐relevant fabrication method is challenging. Ideally, industrial‐scale fabrication of inorganic perovskite devices would entail fabrication processes involving low‐temperature steps (below 150 °C) in an ambient room environment with a short annealing time (preferably a few minutes), promoting low‐cost processes and suitability for continuous high‐throughput manufacturing via roll‐to‐roll fabrication on flexible substrates, such as PET or polyimide. To understand the potential and challenges of transitioning the fabrication of inorganic PSCs toward such a manufacturing method, the following sections delve into the effects of moisture, oxygen, and temperature on forming inorganic perovskite films.

### 4.1 Influence of Environmental Factors on Inorganic Perovskite films

The environment is one of the factors influencing film deposition and annealing. High‐efficiency CsPbI_3_ devices require film fabrication in an inert gas‐filled glovebox because black CsPbI_3_ phases and Cs halide precursors are sensitive to moisture, which can trigger phase change and ultimately affect the efficiency of the final device. Hence, inorganic perovskites are generally fabricated in an inert environment. Some studies have conducted film fabrication in an environment with controlled humidity levels.^[^ ^179^, ^180^, ^181^, ^182^ ^]^ Despite significant progress, fabricating perovskite films in an ambient atmosphere remains a challenging task,^[^ ^165^, ^181^, ^182^, ^183^, ^184^ ^]^ as the device efficiencies remain slightly inferior to those manufactured in an inert environment, with an efficiency of 21.4% achieved under 30% relative humidity (RH) through additive engineering and annealing the films at 200 °C.^[^ ^165^, ^183^, ^185^, ^186^ ^]^ Several studies have demonstrated that annealing perovskite films in an ambient atmosphere can improve crystallization and grain growth.^[^ ^183^, ^186^, ^187^, ^188^, ^189^ ^]^ Several researchers have deposited their films in an inert atmosphere before transferring them to an ambient environment for prolonged annealing.

**Table** **1** provides a comprehensive summary of the ambient‐processed films, the strategies utilized, and the corresponding performances of the devices constructed. It is worth noting that while entirely ambient processed perovskites are currently associated with slightly lower device efficiencies, they hold significant potential for low‐cost upscaling. The following section delves into the effects of moisture, oxygen, and temperature on fabricating inorganic perovskites, offering insights that could further enhance the potential of ambient processing.

**Table 1:** Summary of the ambient‐processed inorganic PSCs and their synthetic conditions, architecture, performance, and stability. The table is ascendingly sorted based on the annealing temperature.

Perovskite	Additive/strategy	Phase	Processing conditions		Architecture	Active area [cm^2^]	*V*_oc_ [V]	*J*_sc_ [mA cm^−2^]	FF [%]	PCE [%]	Refs.
			Relative humidity [RH, %]	Annealing temp. [°C]/duration [min]							
CsPbI_3_	QDs; dopant‐free HTMs	α	<30	N/A	ITO/c‐TiO_2_/CsPbI_3_‐QDs/PTB7/MoO_3_/Ag	0.073	1.27	12.4	80.0	12.6	[144]
	QDs; MeOAc as antisolvent	α	<10	N/A	ITO/SnO_2_/PCBM:CsPbI_3_/CsPbI_3_‐QDs/PTB7/MoO_3_/Ag PET/ITO/SnO_2_/PCBM:CsPbI_3_/CsPbI_3_‐QDs/PTB7/MoO_3_/Ag	0.072	1.26 1.24	15.2 13.6	78.0 73.0	15.1 12.3	[145]
	HI	γ	<30	100 dipped in IPA, 10 100, 4	FTO/c‐TiO_2_/CsPbI_3_/spiro‐OMeTAD/Ag	0.12	0.66	11.9	52.5	4.13	[146]
	HPbI_3_, antihot	γ	30	170	FTO/TiO_2_/CsPbI_3_/PTAA/Au	N.M	1.11	18.3	78.0	15.9	[147]
	MXene, DMAI; ultrasonic spraying	γ	20	170	FTO/PTAA/CsPbI_3_/OMXene‐CsPbI_3_/CPTA/BCP/Ag	0.096	1.21	19.9	82.0	19.69	[148]
	The light‐soaking effect, DMAPbI_3_	γ	<22	180, 15	FTO/c‐TiO_2_/CsPbI_3_/spiro‐OMeTAD/Au	0.12	1.16	19.2	82.5	18.3	[149]
	4‐Aminothiophenol (4‐ATP), DMAI	β	<10	180, 20	FTO/c‐TiO_2_/CsPbI_3_/spiro‐OMeTAD/Au	0.1	1.18	20.4	83.5	20.1	[150]
	Perhydropolysilazane (PHPS)/MeOAc antisolvent template, HPbI_3_	γ	60	180, 4–5	FTO/c‐TiO_2_/CsPbI_3_:PHPS/spiro‐OMeTAD/Au	N.M^a)^	1.18	20.2	80.6	19.2	[151]
	Zn(C_6_F_5_)_2_ and airflow‐assisted blade coating	γ	≈35	180, 20	FTO/SnO_2_/CsPbI_3_:Zn(C_6_F_5_)_2_/CsPbI_3_/spiro‐OMeTAD/Au	0.09	1.12	20.7	82.0	19.0	[152]
	EMIMHSO_4_, HPbI_3_, PbBr_2_	γ	N.M	180, 24	FTO/c‐TiO_2_/CsPbI_3_:EMIMHSO_4_/spiro‐OMeTAD/Au	0.09 1.0	1.17 1.12	20.6 19.5	83.0 66.8	20.0 14.6	[153]
	1,4‐Butanediamine (DAB) surface treatment, 3,5‐difluorobenzoicacidhydrazide (FBJ) additive, DMAI	γ	35	185, 10	ITO/P3CT‐N/CsPbI_3_/PCBM/C60/BCP/Ag	N.M^a)^	1.21	20.4	80.4	19.8	[154]
	MMDS, DMAI	β	40	190, 10	ITO/P3CT‐N/CsPbI_3_/PCBM/C60/TPBi/Cu	0.09	1.17	20.6	78.8	19.0	[155]
	HPbI_3_	α	10–20	200, 10	FTO/c‐TiO_2_/m‐TiO_2_/CsPbI_3_/carbon	0.0625	0.79	18.5	65.0	9.5	[156]
	FACl/IPA intermediate treatment; HPbI_3_	γ	<30	60, 5 200, 5	ITO/OEDOT:PSS/CsPbI_3_/PCBM/BCP/Ag	N.M^a)^	1.02	19.1	79.2	15.5	[157]
	1,5‐Pentanediamine (PDA) passivation	β	≈30	200, 5	FTO/P3CT‐N/CsPbI_3_:PDA/PCBM/BCP/Ag	N.M	1.15	20.4	83.2	19.5	[158]
	MAAD, DAB surface treatment, HPbI_3_	β	0–60	200, 5–7	ITO/P3CT‐N/CsPbI_3_/PCBM/C60/BCP/Ag	0.09	1.16	20.5	81.1	19.3	[159]
	3,5‐Difluorobenzoic acid hydrazide (FBJ) DMAI	γ	35	200, 8	ITO/P3CT‐N/CsPbI_3_/PCBM/C60/BCP/Ag	N.M^a)^	1.23	20.3	77.4	19.3	[160]
	Ethacridine lactate additive; HPbI_3_	γ	≈30	200, 2	ITO/PEDOT:PSS/Me‐4PACz/CsPbI_3_:EAL/PCBM/C60/BCP/Ag	0.05	1.20	21.5	81.6	21.1	[161]
	Me‐4PACz and EAL additive; HPbI_3_	γ	≈30	200, 2	ITO/PEDOT:PSS/Me‐4PACz/CsPbI_3_:Me‐4PACz/PCBM/C60/BCP/Ag	0.05	1.21	21.5	82.2	21.4	[162]
	DMAI, PTACl	β	20–85	150, 5 210, 5	FTO/SnO_2_/CsPbI_3_/PTACl/spiro‐OMeTAD/MoO_3_/Ag	0.05	1.18	20.5	78.1	18.9	[163]
	DMAI, Zn(C_6_F_5_)_2_ Phase heterojunction GAI‐assisted coevaporation	β γ	25–30	60, 5 100, 10 210, 20	FTO/c‐TiO_2_/SnO_2_/β‐γ‐CsPbI_3_/PC_61_BM/P3HT‐SMe‐TATPyr/Ag	0.09	1.22	21.7	81.5	21.6	[22]
	Excess DMAI, PTACl	β	<10	210, 5	FTO/c‐TiO_2_/CsPbI_3_/PTACl/spiro‐OMeTAD//Ag	0.10	1.14	20.2	82.7	19.0	[164]
	DMAI, MACl, OAI	β	15–30	210, 5	FTO/TiO_2_/CsPbI_3_:MACl/OAI/spiro‐OMeTAD/Au	0.094	1.20	20.6	82.5	20.4	[165]
	DMSO as a solvent, DMAI, vacuum drying	γ	<15	Vacuum treatment (≈0.1 MPa) 220, 5	FTO/c‐TiO_2_/CsPbI_3_/carbon	0.0625 1.0	1.10 1.15	19.6 18.9	77.0 52.9	16.7 11.5	[166]
CsPbI_3‐_ *_x_* Br*_x_*	HOCl_3_ additive, excess DMAI	γ	N.M	220, 5	FTO/c‐TiO_2_/mp‐TiO_2_/CsPbI_2.5_Br_0.5_/P3HT/Au	0.09	1.27	19.5	79.0	19.6	[122]
	In situ hot oxygen cleansing and passivation, DMAPbI_3_	γ	10–20	210, 5	FTO/TiO_2_/Î^2^‐CsPbI_2.85_Br_0.149_Cl_0.001_/spiro‐OMeTAD/Au	0.09	1.23	19.9	80.1	19.7	[167]
	Methylammonium acetate (MAAC) solvent	γ	N.M	150, 2 250, 3 350, 5	FTO/SnO_2_/CsPbI_2.5_Br_0.5_/spiro‐OMeTAD/MoO_3_/Ag	0.05	1.30	17.7	74.2	17.1	[168]
CsPbI_2_Br	Phthalimide additive, doctor blading	γ	N.M	200, 10	FTO/c‐TiO_2_/CsPbI_2_Br/carbon	0.09	1.3	15.8	68.0	14.0	[54]
	CsPb_2_I_4_Br/CsPbI_2_Br bulk heterojunction (BHJ)	α	30–70	270, 10	FTO/c‐TiO_2_/CsPbI_2_Br/CsPb_2_I_4_Br/carbon FTO/c‐TiO_2_/CsPbI_2_Br/CsPb_2_I_4_Br/spiro‐OMeTAD/Au	0.125	1.32 1.36	14.6 15.5	79.1 80.9	15.3 17.0	[169]
	A‐site doping (Rb^+^); hot‐air blowing	α	N.M	280, 10	FTO/c‐TiO_2_/mp‐TiO_2_/Cs_0.99_Rb_0.01_PbI_2_Br/P3HT/Au	0.09	1.32	16.3	80.0	17.2	[125]
	SmI_3_ doping	α	N.M	280, 10	FTO/c‐TiO_2_/m‐TiO_2_/CsPb_0.97_Sm_0.03_I_2_Br/P3HT/Au	0.09	1.30	15.9	76.0	15.7	[126]
	EuI_2_ doping	α	<5%	25, 5 280, 10	TiO_2_/c‐TiO2/m‐TiO_2_/CsPb_0.95_Eu_0.05_I_2_Br/spiro‐OMeTAD/Au	0.16	1.22	14.6	76.6	13.7	[127]
CsPbIBr_2_	Sn‐element doping, SnBr_2_	γ	<90	80, 30 350, 10	FTO/c‐TiO_2_/m‐TiO_2_/CsPb_0.9_Sn_0.1_IBr_2_/carbon	0.09	1.26	14.3	63.0	11.3	[128]
	Cosensitization with 5,5‐bis(2,6‐dioctoxyphenyl)‐10‐(bis(4‐hexylphenyl)‐amino‐20‐4‐carboxyphenylethynyl)porphyrinato]zinc(II) (YD_2_‐o‐C_8_)	γ	≈35	100, 10 180, 60	ITO/SnO_2_/SnCl_2_/CsPbIBr_2_/YD_2_‐o‐C_8_/spiro‐OMeTAD/Au	N.M^a)^	1.37	12.1	61.0	10.1	[170]
	InI_3_	α	N.M	320, 10	FTO/c‐TiO_2_/m‐TiO_2_/CsPb_0.85_In_0.15_I_1.45_Br_1.55_/carbon	0.09	1.20	15.7	64.0	12.0	[100]
CsPbBr_3_	Muscovite substrate, 2‐step deposition	α	<30	100, 40 250, 5	Muscovite/ITO/Sb‐TiO_2_/CsPbBr_3_/carbon	0.04	1.53	4.79	77.9	5.71	[171]
	2‐Step deposition; alkyl acid‐capped QDs as HTM and long persistence phosphor (LPP)‐modified carbon as electrode	γ	w/o control	90 for PbBr_2_, 60 250 for CsBr, 5	FTO/c‐TiO_2_/m‐TiO_2_/CsPbBr_3_/C12‐alkyl acid capped CuInS_2_/ZnS QD/LPP‐carbon	0.09	1.63	7.73	86.3	10.9	[76]
	Low‐boiling point ligands and hexane/IPA for synthesis; QDs	α	N.M	25, 5	FTO/c‐TiO_2_/CsPbBr_3_/spiro‐OMeTAD/Au	0.094	1.54	5.65	62.4	5.42	[172]
	Guanidinium thiocyanate (GASCN) post‐treatment; QDs	α	<10	25, N.M	FTO/c‐TiO_2_/CsPbBr_3_/GASCN/PTAA/MoO_3_/Ag	0.073	1.54	4.49	72.5	5.01	[173]
	Two‐step solution process	N.M^a)^	N.M^a)^	70, 30 250, 10	FTO/c‐TiO_2_/m‐TiO_2_/CsPbBr_3_/PTAA/Au	0.24	1.25	6.70	73.0	6.20	[174]
	Chlorine doping	N.M^a)^	N.M^a)^	70, 30 250, 10	FTO/c‐TiO_2_/CsPbBr_3_–Cl/spiro‐OMeTAD/Ag	0.09	1.02	8.47	71.6	6.21	[175]
	Thiocyanate ethyl acetate treatment, QDs	N.M^a)^	N.M^a)^	25, N.M	FTO/ZnO/CsPbBr_3_–CsPb_2_Br_5_/spiro‐OMeTAD/Au	0.09	1.43	6.17	77.2	6.81	[176]
	TiO_2_/SnO_2_ bilayer; multistep spin‐coating	α	N.M^a)^	90, 60 250, 5	FTO/c‐TiO_2_/SnO_2_/CsPbBr_3_/CuPc/C	0.07 1.0	1.31 1.4	8.24 6.93	81.4 71.3	8.79 6.90	[97]
	Multistep sequential dual‐source vacuum deposition	α	0–50	N.M, 30	FTO/c‐TiO_2_/CsPbBr_3_/carbon	N.M	1.522	7.24	80.4	8.86	[177]
	Morphology control, two‐step annealing–‐IPA antisolvent	α	20–50	70, 70 90, 15 250, 5	FTO/c‐TiO_2_/CsPbBr_3_/carbon	0.09	1.49	6.89	79.0	8.11	[178]

^a)^ Not mentioned in the literature. Inert ‐ glovebox; QD ‐ quantum dot.

#### 4.1.1 Influence of Moisture

**Figure 6:**
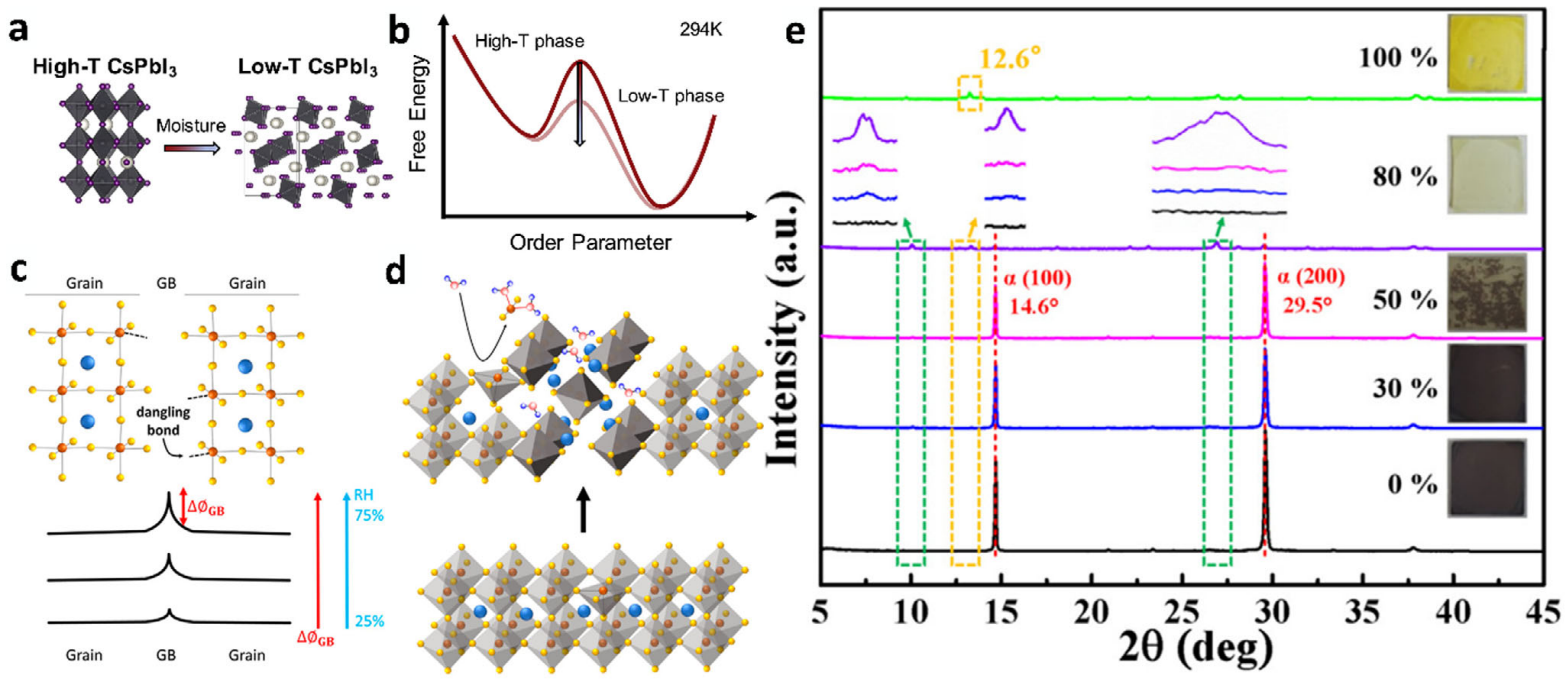
a) Phase transformation of CsPbI_3_ due to moisture and b) a visual representation of the energy diagram of CsPbI_3_ (solid red line) compared to the typical humidity‐induced alteration (pale red line) of the energy scheme. Reproduced with permission.^[^ ^198^ ^]^ Copyright 2021, Elsevier. c,d) Schematic showing the presence of undercoordinated Pb^2+^ cations at grain boundaries under humidity conditions and potential difference: degradation of CsPbBr_3_ into byproduct (Cs_4_PbBr_6_) in the presence of water molecules. Reproduced with permission.^[^ ^199^ ^]^ Copyright 2022, American Chemical Society. e) XRD patterns of CsPbI_2_Br films were recorded under 0%, 30%, 50%, 80%, and 100% RH for 10 min, and the respective images of the samples. Reproduced with permission.^[^ ^200^ ^]^ Copyright 2022, American Chemical Society.

**Figure 7:**
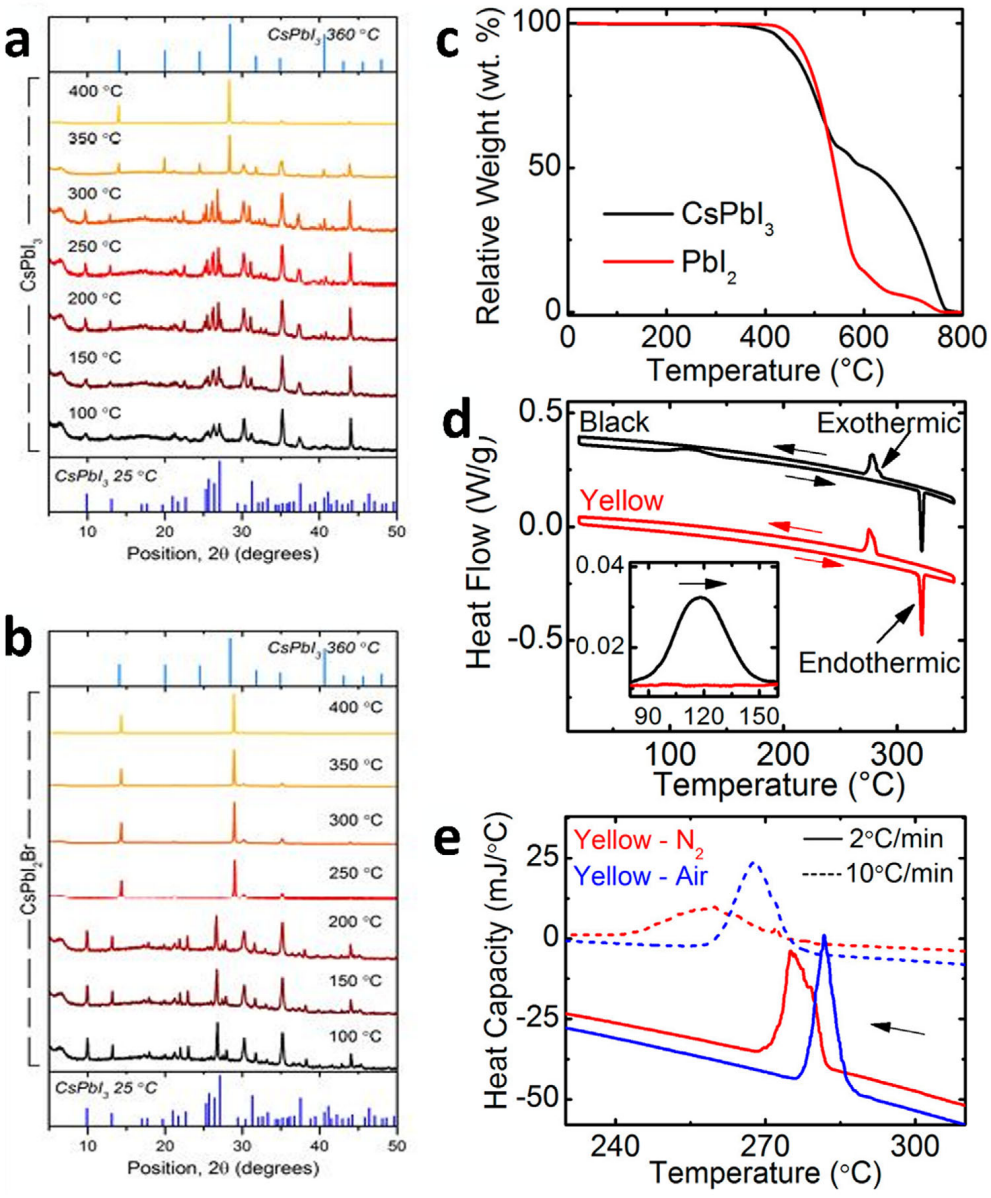
In situ annealing XRD patterns of a) CsPbI_3_ and b) CsPbI_2_Br films on glass, along with the reference patterns. Reproduced with permission.^[^ ^87^ ^]^ Copyright 2016, John Wiley and Sons. c) TGA curves for PbI_2_ and CsPbI_3_ powder samples. d) DSC curves for CsPbI_3_ powders. e) Humidity and cooling rate effects on phase δ‐CsPbI_3_. Reproduced with permission.^[^ ^231^ ^]^ Copyright 2017, American Chemical Society.

Despite fabricating at high temperatures (typically over 200 °C annealing), CsPbX_3_ films in the resulting black phase (α, β, or γ phase) are prone to an undesired phase transition to a low‐temperature non‐perovskite yellow δ‐phase, particularly under humid ambient conditions.^[^ ^190^, ^191^, ^192^, ^193^ ^]^ Straus et al. synthesized CsPbI_3_ single crystals and studied the phase transition using powder XRD under continuous gas flow, resulting in a stable black γ‐phase in dry Ar and dry O atmospheres at room temperature (**Figures** **6** and **7**). However, the phase catalytically converted to a yellow δ‐phase in the presence of moisture.^[^ ^194^ ^]^ In devices, polycrystalline films can undergo a phase transition with environmental strain due to grain boundaries, lattice vacancies, and lowered energy barriers to nucleation (Figure 6a,b).^[^ ^195^ ^]^ To achieve the metastable black α‐phase at room temperature, Dastidar et al. conducted an experiment where they rapidly cooled the high‐temperature α‐phase and maintained it at room temperature in an inert N_2_‐glovebox to avoid moisture effects.^[^ ^196^ ^]^ The observed kinetics of the black‐to‐yellow conversion displayed a catalytic impact rather than a change in equilibrium due to water adduct formation, as observed in hybrid perovskites.^[^ ^197^ ^]^

Molecular dynamics simulations^[^ ^198^ ^]^ show that a thin layer of water in contact with a high‐temperature CsPbI_3_ perovskite phase surface enhances the halide vacancies at the interface of the water and perovskite. The phenomenon is attributed to the high solvation enthalpy of halide ions and their low vacancy formation energies (Figure 6c,d). Halide vacancies act as defect sites, reducing the phase‐transition barrier between the metastable black high‐temperature γ‐phase and the stable yellow low‐temperature non‐perovskite δ‐phase of CsPbI_3_, promoting transformation. Once a single layer of water is present on the surface of high‐temperature CsPbI_3_, the equilibrium concentration of vacancies increases significantly with RH (Figure 6e). Concentration correlates with a higher heterogeneous nucleation rate than the homogeneous rate in a basic nucleation model. Consequently, increased humidity levels are anticipated to rapidly increase the nucleation rate of δ‐phase CsPbI_3_. However, no crystallographic studies have examined the impact of incremental increases in ambient moisture and the effects on the phase transition of CsPbI_3_, particularly in the range from 20% to 80% RH. Previous studies offer valuable insights into the underlying relationships within CsPbI_3_.

Researchers have explored various strategies to stabilize the black phase of CsPbI_3_ under humid conditions, focusing on tuning the tolerance factors, crystal grain size reduction, and surface passivation. Additives such as HI or HPbI_3_ and DMA or DMAPbI_3_ are mostly used to stabilize the black phase. However, some strategies reduce photovoltaic performance while stabilizing the black phase. Specifically, phase stabilizing methods, including anionic modification by partially replacing I^−^ with Br^−^, weaken the light‐harvesting ability and narrow the absorption spectra.^[^ ^179^, ^180^, ^181^ ^]^ Other strategies, including incorporating quasi‐2D perovskite capping layers, hinder carrier transport by introducing organic ligand passivation layers with anchored functional groups, reducing the grain size of inorganic perovskites, and exacerbating the volatility of organic components at increased temperatures, as observed for organic–inorganic hybrid perovskites.^[^ ^201^, ^202^ ^]^

Researchers have comprehensively studied the phase stabilization of CsPbI_3_ perovskites in the air using organic molecule passivation engineering and crystal growth regulation strategies.^[^ ^156^, ^203^, ^204^ ^]^ For example, some researchers prepared a uniform and dense perovskite layer by facilitating the transition of the CsPbI_3_ intermediate phase using sequential dripping of a methylammonium chloride solution combined with surface passivation using octyl ammonium iodides (OAI) with strictly controlled RH (below 30%).^[^ ^205^ ^]^ Fang et al. added maleic anhydride molecules to perovskite precursors to broaden the humidity operating window in high‐humidity environments.^[^ ^206^ ^]^ Another group of researchers applied a synergetic method of preheating the substrate and spraying antisolvent methyl acetate (MeOAc) to accelerate the precursor concentrations up to supersaturation and heterogeneous nucleation.^[^ ^147^ ^]^

Previous studies have demonstrated that the effect of undesired moisture on perovskite films can be suppressed by reducing the exposure time of the nucleation phase to high humidity and accelerating the growth of the intermediate phase during film formation. Wang et al. discovered the impact of water on CsPbI_3_ perovskites.^[^ ^207^ ^]^ Contrary to popular belief, they found that preparing CsPbI_3_ in a humid atmosphere rather than in a dry or inert atmosphere resulted in higher film quality and device performance. Smaller colloidal particles comprising low‐I coordination complexes formed when H_2_O was added to the precursor solution. The process slowed the crystallization of the DMAPbI_3_ intermediate, which in turn accelerated the conversion of DMAPbI_3_ to CsPbI_3_, resulting in better crystallinity of the black‐phase CsPbI_3_ and improved orientation of the CsPbI_3_ crystals. Notably, the film fabrication process still involved spin coating and annealing at 220 °C in dry air, RH < 1%, and in a glovebox.

Highly explored CsPbX_3_ QDs/nanocrystals are typically synthesized in a vacuum and dispersed in nonpolar solvents. However, film deposition and annealing of the inks are mainly performed in an ambient environment with RH < 30%.^[^ ^29^, ^173^, ^208^, ^209^, ^210^, ^211^, ^212^, ^213^ ^]^ Cheng et al.^[^ ^214^ ^]^ detailed investigation of the effects of water on inorganic perovskites illustrated that even a trace amount of water on CsPbX_3_ (X = I^−^ or Br^−^) caused conversions from Cs_4_PbX_6_ to CsPbX_3_ or from CsPbX_3_ to CsPb_2_X_5_.^[^ ^215^, ^216^ ^]^ However, high humidity levels led to the breakdown of inorganic perovskites into PbX_2_ and CsX via interactions with aggregated water, resulting in partial dissolution. Furthermore, the chemical reactions could be reversed in some instances when water was removed from the system.

Snaith and his team thoroughly investigated the performance of CsPbI*_x_* Br_3−_ *_x_* films by varying the I^−^/Br^−^ ratio.^[^ ^87^ ^]^ They found that increasing the amount of Br^−^ in the film resulted in a broader bandgap and improved stability under varying thermal and humidity environments. Recently, Ma et al. analyzed the effects of halide mixing (I, Br, and Cl) on the crystallization and phase evolution of CsPbX_3_ perovskites.^[^ ^217^ ^]^ They utilized in situ grazing‐incidence wide‐angle X‐ray scattering and discovered that Br helped create a black perovskite phase while preventing the transition to the yellow non‐perovskite phase. Although mixed anion or Cs, Pb, and Br perovskites are relatively stable, moisture still affects them. When exposed to water molecules, hybrid perovskites form PbI_2_·CsPbI_2_Br, which, on exposure to humid conditions, forms δ‐phase compounds (Figure 6e), similar to CsPbI_3_.^[^ ^200^ ^]^ However, XRD analysis shows that the formation of the δ‐phase can be reversed by regulating temperature and removing water molecules.

Further, Zhang et al. revealed that a CsPbI_2_Br film fabricated in a glovebox for reannealing in dry air (H_2_O < 0.1 ppm) underwent an oxidative decomposition reaction, resulting in the formation of CsPbIBr_2_ and PbO instead of phase degradation.^[^ ^218^ ^]^ Moderate moisture (30–60% RH) reannealing significantly slowed the oxidation process and facilitated a passivating effect at the grain boundaries. Thus, band coupling occurred between CsPbI_2_Br and CsPbIBr_2_ during the gradual and orderly oxidation process, which improved charge transfer and reduced voltage loss. The improvements led to a higher *V*_oc_ of 1.32 V compared to the initial value of 1.05 V. Liu and colleagues utilized angle‐resolved X‐ray photoelectron spectroscopy to investigate the formation of PbO. Their findings revealed that water molecules adhered only to the lattice surface of CsPbI_2_Br, a phenomenon distinct from hydrolysis observed in organic–inorganic hybrid PSCs.

The absorption of water molecules on the lattice surface leads to the migration of halide ions toward the surface owing to their high hydration enthalpy. The migration results in halide vacancies within the lattice, lowering the energy barrier for phase transition from α‐ to δ‐phase. Thus, exposure to water vapor prompts the transformation of the CsPbI_2_Br film from α‐ to δ‐phase, although phase transition can be partially reversed through subsequent annealing.^[^ ^219^ ^]^

Huang et al. used scanning probe microscopy to investigate the surface potential of CsPbBr_3_.^[^ ^199^ ^]^ They found a correlation between higher humidity levels, increased surface potentials, and decreased work function (Figure 6c,d). Their findings suggested that the number of detected electrons increased with humidity. In the typical process of creating CsPbBr_3_ films from CsBr and PbBr_2_, CsPbBr_3_, 0D Cs_4_PbBr_6_, and 2D CsPb_2_Br_5_ were formed because of their similar formation enthalpies.^[^ ^220^, ^221^ ^]^

In a humid environment, water molecules can affect Cs_4_PbBr_6_, causing CsBr to leach from the crystal. Transformation converts Cs_4_PbBr_6_ into CsPbBr_3_ while maintaining the PbBr_6_ octahedron framework (Equation (3)). Subsequent interactions with water vapor remove CsBr, changing the coordination number of Pb(II) from six to eight. The change in the Pb–Br coordination environment ultimately causes the decomposition of the CsPbBr_3_ crystal into an amorphous state. The amorphous phase transforms into a CsPb_2_Br_5_ crystal because of its inherent instability, as shown in Equation (4)

(3) $$Cs_{4}\mathrm{PbB}r_{6}\overset{H_{2}O}{\to }\;\;\mathrm{CsPbB}r_{3}+3\mathrm{CsBr}$$

(4) $$2\mathrm{CsPbB}r_{3}\overset{H_{2}O}{\to }\mathrm{CsP}b_{2}Br_{5}+\mathrm{CsBr}$$

A recent study conducted by Chen and co‐workers^[^ ^222^ ^]^ indicated that treating CsPbBr_3_ devices with water vapor significantly enhanced their crystal quality and optical properties. Water vapor treatment removed CsBr from the lattice, transforming the phase into lower‐dimensional perovskites (Equation (2)). However, post‐annealing boosted ion migration at the grain boundaries and increased grain sizes, reducing defect density. Consequently, HTL‐free devices achieved a PCE of 7.45%.

Nevertheless, rapid crystal growth often results in low coverage and poor perovskite film morphology, leading to high trap concentrations that reduce the efficiency and stability of PSCs. Moreover, the volatility of organic molecules used as additives and passivating agents poses a challenge to the thermal stability of PSCs. Therefore, solving the challenges of fast deposition and poor film quality is crucial to achieving high‐performance CsPbI_3_ PSCs under ambient conditions.

#### 4.1.2 Influence of Oxygen

Snaith and co‐workers highlighted that incorporating HI as an additive formed a CsPbI_3_ black phase and demonstrated that the phase maintained its stability when not exposed to air.^[^ ^13^ ^]^ Tsvetkov et al. used a Calvet‐type microcalorimeter to determine the precursor and CsPbX_3_ solution enthalpies and investigate the extrinsic stability of the materials.^[^ ^223^ ^]^ They calculated the Gibbs free energy change and correlated the two values with potential decomposition pathways in the presence of O_2_. Their results indicated that CsPbI_3_ was more likely to degrade in the presence of O_2_ compared to CsPbBr_3_ or CsPbCl_3_ (CsPbI_3_ > CsPbBr_3_ > CsPbCl_3_). Further, they suggested the potential to impede the degradation process through kinetic interventions, highlighting the requirement for further research in this area.

To ensure the preservation of the desired black α‐CsPbI_3_ perovskite phase in ambient air environments, Luo et al. implemented sequential solvent engineering. The technique involved adding HI, dipping the films into a hot IPA solution, and annealing at 100 °C in the air.^[^ ^183^ ^]^ Swarnkar et al. stabilized the cubic phase by creating QD films in ambient air that remained stable for several months.^[^ ^224^ ^]^ Their research highlighted the potential of bandgap tuning (585 and 670 nm) via size control of QDs. Yuan et al. built on this foundation by developing an innovative approach for dopant‐free polymeric HTLs, achieving an efficiency of 12.55% in CsPbI_3_ QD solar cells.^[^ ^144^ ^]^ Their work with dopant‐free polymeric HTLs facilitated efficient charge extraction at QD/polymer interfaces.

Kweon et al.^[^ ^225^ ^]^ discovered that during the phase transition of CsPbX_3_, the bonds were rearranged in the PbX_6_ octahedra. The rearrangement converted corner‐sharing PbX_6_ in the γ‐phase into edge‐sharing PbX_6_ in the δ‐phase. Bond rearrangement in local PbX_6_ octahedra promoted the nucleation of the δ‐phase in the γ‐phase. Enthalpy calculations showed that the transformation energy barrier decreased significantly in moisture owing to the stabilization of undercoordinated Pb atoms. The results were achieved by forming an extra Pb─O bond during PbX_6_ bond rearrangement, suggesting that moisture‐initiated bond reconfiguration in adjacent PbX_6_ octahedra in the γ‐phase, leading to easy nucleation of the δ‐phase. However, the negligible change in the energy barrier in the presence of O_2_ or N_2_ indicated that these gas molecules were unlikely to affect the nucleation process. Positive molecular insertion energies were discovered for N_2_, O_2_, and Ar in both γ‐ and δ‐phases. Therefore, the insertion of these molecules was unfavorable from an energetic perspective. However, integrating these molecules into the γ‐phase reduced the thermodynamic driving force for phase transformation while maintaining the intrinsic activation energy.

The aforementioned discoveries have opened several avenues for further research on material synthesis or treatment in controlled ambient environments at high and low temperatures. Yoon et al. achieved a PCE of 20.37% with β‐CsPbI_3_ PSCs through surface engineering of perovskite films with MACl annealed at 210 °C, 15–30% RH.^[^ ^226^ ^]^ Similarly, Huang et al. found that careful control over stoichiometry and annealing in the atmosphere at 350 °C yielded γ‐CsPbI_3_ solar cells with a PCE of 16.3%.^[^ ^227^ ^]^ Additionally, research has shown that additives can effectively modulate perovskite crystallization dynamics and produce high‐quality perovskite films. Iqbal et al. studied the impact of a perovskite film annealing environment and discovered that atmospheric annealing of glovebox‐processed CsPbI_3_ films resulted in fewer defects and improved band alignment.^[^ ^188^ ^]^ During the study, the films were initially annealed at 210 °C under 1% RH and then at over 20% RH, resulting in 19.8% device efficiency. Wang et al. studied the role of DMAI in controlling crystallization and developed black‐phase CsPbI_3_ perovskite films at 150 °C, 5–10 % RH to achieve solar cell efficiencies up to 19%.^[^ ^228^ ^]^ Duan et al. added formamidine acetate to the precursor solution, gaining over 18% efficiency with γ‐CsPbI_3_ films fabricated at 340 °C, 10% RH, improving their phase purity and electronic quality.^[^ ^229^ ^]^

As experimentally emphasized by Liu et al., compared to hybrid PSCs, inorganic halide perovskites can be positively impacted by oxygen with passivation while mitigating its adverse effects.^[^ ^230^ ^]^ This is because the inorganic cations lack acid protons, making them ideal candidates for O_2_ passivation in perovskites. Additionally, Cs‐based inorganic perovskites (CsPbX_3_, X = Cl, Br, I) exhibited better thermal stability than hybrid perovskites because Cs^+^ replaced volatile organic cations, allowing compositional stability even at 500 °C.^[^ ^25^, ^231^, ^232^ ^]^

Moot et al.^[^ ^233^ ^]^ reported that CsPbI_3_ nanocrystals were prone to photooxidative degradation. The degradation could be prevented by using encapsulants such as polystyrene or O_2_‐scavenging charge‐transport layers. Although polycrystalline CsPbI_3_ is typically unstable, nanocrystalline films are more stable and retain their perovskite phases. However, Moot et al. found that the film underwent slow surface‐mediated photooxidation bleaching due to reactive O_2_ species. Bleaching occurred when the ligands were deprotonated, I evolved, and metal carbonate species were formed.^[^ ^233^ ^]^ For prevention, maintaining native oleylammonium as the amine ligand or using terminating ligand salts containing bromides, such as FABr, PTABr, or TMA, was recommended. The ligands reduced the number of volatile, undercoordinated sites that contributed significantly to photooxidative degradation. According to DFT studies by Tsvetkov et al.,^[^ ^234^ ^]^ the stability of CsPbI_3_ was highly dependent on the presence of gaseous O_2_, H_2_O, and CO_2_, whereas CsPbCl_3_ and CsPbBr_3_ were less sensitive to interactions with the ambient atmosphere. However, according to the aforementioned studies, all types of CsPbX_3_ degrade in the presence of liquid water.

#### 4.1.3 Temperature

The conventional method of producing high‐performance black‐phase CsPbX_3_ involves high temperatures (>310 °C). However, research has made significant progress by developing several alternative techniques for obtaining highly pure photoactive perovskite‐phase CsPbX_3_ thin films suitable for low‐temperature manufacturing processes. Specifically, HI‐assisted methods reduce the temperature of black‐phase, stable, inorganic perovskites. Snaith and co‐workers pioneered the development of inorganic CsPbI_3_ PSCs, marking a significant step forward in the field.^[^ ^15^ ^]^ Their research demonstrated that δ‐CsPbI_3_ maintained stability at room temperature while the black α‐phase only crystallized at temperatures exceeding 310 °C (Figure 7). Introducing HI as an additive converted the yellow δ‐CsPbI_3_ phase to the black α‐phase at a modest temperature of 100 °C. The resulting bandgap (1.73 eV) yielded efficiencies of 2.9% and 1.9% in n–i–p and p–i–n cell architectures, respectively. Luo et al. modified the method and treated excess HI‐assisted fabricated films by dipping them in IPA after spin coating. Their findings revealed a new low‐temperature phase transition, forming an intermediate Cs_4_PbI_6_ phase (Equations (5)–(7)) and stabilizing α‐CsPbI_3_ at low temperatures. The resulting PSCs exhibited a relatively higher PCE of 4.13% compared to the prior study by Snaith and co‐workers^[^ ^15^, ^183^ ^]^

(5) $$4\mathrm{CsI}+\mathrm{Pb}I_{2}\to Cs_{4}\mathrm{Pb}I_{6}$$

After IPA treatment

(6) $$Cs_{4}\mathrm{Pb}I_{6}\to \alpha -\mathrm{CsPb}I_{3}+3\mathrm{CsI}$$

(7) $$Cs_{4}\mathrm{Pb}I_{6}+3\mathrm{Pb}I_{2}\to 4\alpha -\mathrm{CsPb}I_{3}$$

Research on the impact of pH on perovskite crystallization^[^ ^235^ ^]^ has suggested that acidic substances react with DMF, creating formic acid and dimethylamine. Thus, inorganic acids such as HI and HPbI_3_ do not facilitate crystallization, but the organic compound DMAI does.^[^ ^236^, ^237^ ^]^ Another DMAI‐modified strategy for producing high‐quality black phase γ‐CsPbI_3_ perovskite at only 100 °C involves adding excess CsI and dipping the resulting film in a 2‐propanol solution, which helps to achieve a stoichiometric balance between the DMAPbI_3_ and Cs_4_PbI_6_ intermediates formed.^[^ ^238^ ^]^ Similar approaches have been employed for CsPbI_2_Br perovskite films using a precursor combination of 2CsI + PbBr_2_ + HPbI_3+_ *_x_*.^[^ ^239^ ^]^ The method lowers the crystallization energy barrier for black α‐phase CsPbI_2_Br and produces high‐quality films. Low‐temperature deposition is critical for low‐cost, large‐scale processing, especially for flexible substrates. Multiple methods exist for creating black α‐phase inorganic perovskites at low temperatures, including vacuum deposition, which produces CsPbX_3_ perovskite films. For instance, under Cs‐rich growth conditions, coevaporation of CsI and PbI_2_ precursors leads to synthesizing γ‐CsPbI_3_ at a substrate temperature as low as 50 °C. Another practical approach for improving material stability at lower temperatures involves the formation of perovskite nanocrystals. By reducing the crystallite size, the surface‐to‐volume ratio can be increased, ultimately stabilizing the crystal structure by adjusting the ratio of the surface energy to the total Gibbs energy. However, synthesizing nanocrystals requires various organic components, including solvents and surface ligand combinations. The stoichiometric ratio and alkyl chain length of organic amines and acid‐capping ligands significantly affect the size, shape, and chemical and optoelectronic properties of the resulting perovskite nanocrystals. Organic molecules can participate in the perovskite crystallization process by acting as organic additives or dopants in the precursor solution to tune the formation of the intermediate phase or by modifying the surface and interface of the thin film during crystal growth. The approach has proven effective in passivating defects in the bulk and/or on the surface of materials to tune crystallinity, structure, and energetics at the interfaces.

Huang and co‐workers developed a new method to stabilize α‐CsPbI_3_ films at low temperatures. By incorporating a small amount of sulfobetaine zwitterions as additives into a perovskite solution and utilizing a p–i–n structure, their solar cell achieved an efficiency of 11.4%.^[^ ^240^ ^]^ Additionally, Unold et al. discovered that thermal evaporation effectively produced CsPbI_3_ films at low temperatures. They developed stable γ‐CsPbI_3_ films at 50 °C by controlling the deposition ratios of CsI and PbI_2_, resulting in an efficiency above 12%. Recently, a ternary source coevaporation method using CsI, PbI_2_, and phenylethylammonium iodide was proposed to create stable γ‐CsPbI_3_ films at ≈100 °C, achieving 15% efficiency. Researchers have used various precursor additives such as ionic liquids^[^ ^179^, ^241^, ^242^ ^]^ and multifunctional molecular additives such as Zn(C_6_F_5_)_2_^[^ ^243^ ^]^ to precisely control the growth of perovskite films and hydrophobicity for phase stability under ambient conditions. On using ionic liquids, a device with EMIMHSO_4_‐based CsPbI_3_ films fabricated at 180 °C exhibited a PCE of 20.01%.^[^ ^179^ ^]^ Modification of the precursor with Pb(OAC)_2_ produced CsPbI_3_ films at 100 °C, which resulted in device PCE of 20.17%.^[^ ^244^ ^]^ Similarly, blade coating Zn(C_6_F_5_)_2_‐based perovskites at low temperatures (≤100 °C) led to 19% device efficiency.^[^ ^152^ ^]^

Another practical approach to enhancing stability at room temperature involves halide substitution. Although incorporating bromine into CsPbX_3_ compromises the absorption profile of the iodine‐based CsPbX_3_, it improves thermal stability. In the mixed halide compound CsPbI_2_Br, the orthorhombic‐to‐cubic transition temperature is reduced to 250 °C, compared to 350 °C in CsPbI_3_ (see Figure 7a,b). This decrease is attributed to an increased tolerance factor, crucial in enhancing structural stability.

## 5 State‐of‐the‐Art Inorganic Solar Cells

**Figure 8:**
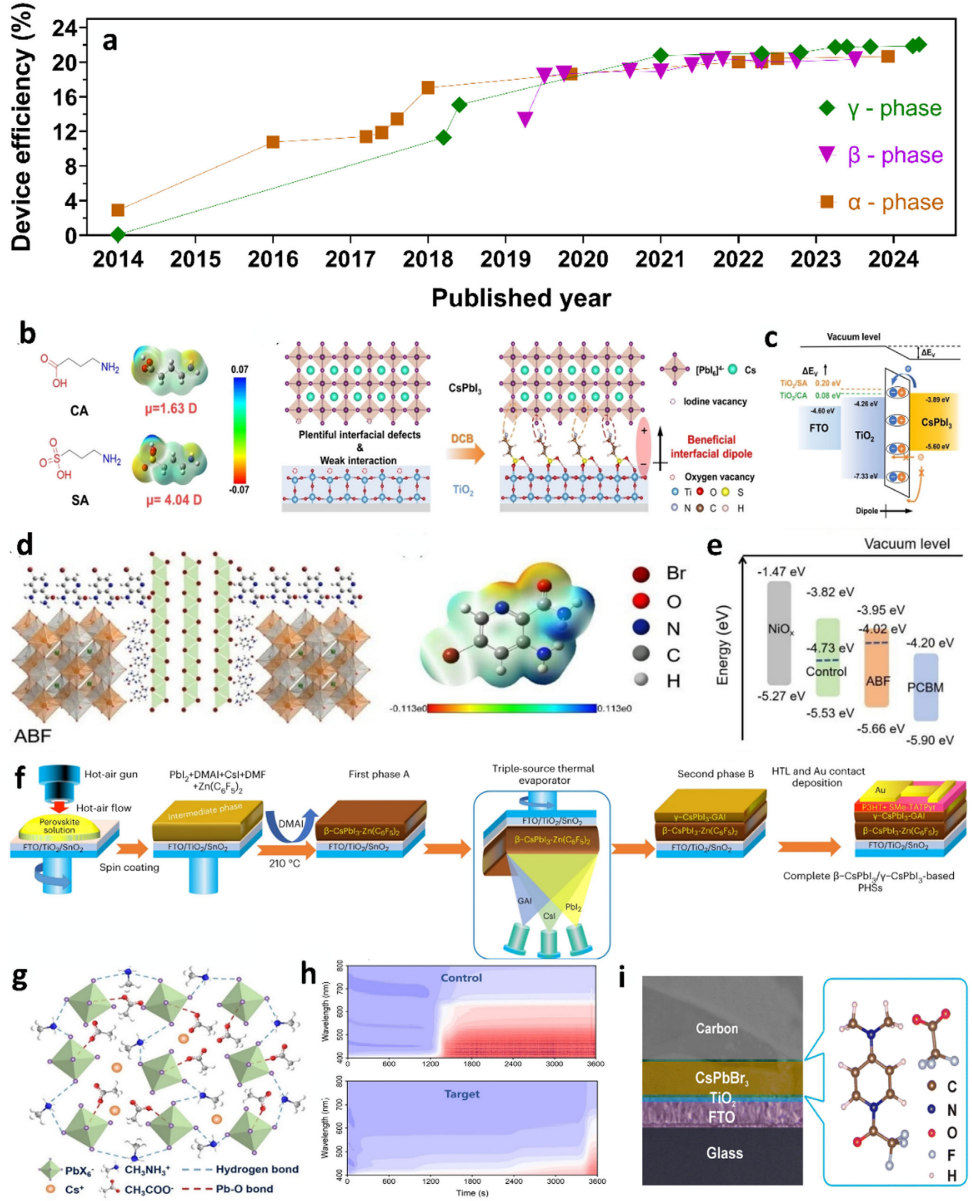
a) Performance evolution of devices constructed with each phase of the CsPbX_3_ perovskite active layers over the years. b–i) State‐of‐the‐art solar cells: the additives or passivation layers used to achieve record performance. (b) Illustration of DCB between the perovskite and TiO_2_ layers follows the molecular structure and electrostatic potential maps for CA and SC molecules. (c) Schematic shows the interfacial dipole of the perovskite/TiO_2_ caused by the DCB. Reproduced with permission.^[^ ^248^ ^]^ Copyright 2024, John Wiley and Sons. (d) Schematic depicting the passivation mechanism in ABF and the electrostatic potential (ESP) images of the ABF molecule. (e) Energy‐level diagram of inverted inorganic PSCs and ABF‐modified perovskite. Reproduced with permission.^[^ ^260^ ^]^ Copyright 2024, John Wiley and Sons. (f) Fabrication pathway to develop perovskite‐based phase‐heterojunction devices: hot‐air processes β‐CsPbI_3_–Zn(C_6_F_5_)_2_ and the triple‐source thermally evaporate γ‐CsPbI_3_–GAI. Phase A denotes the hot‐air‐processed β‐CsPbI_3_–Zn(C_6_F_5_)_2_, and phase B denotes the thermally evaporated γ‐CsPbI_3_–GAI perovskite thin films. The PHS device is completed by depositing the HTL and metal contact. Reproduced with permission.^[^ ^22^ ^]^ Copyright 2023, Springer Nature. (g) Chemical interaction process involved in the crystallization of CsPbI_2_Br in the presence of MAAc. Reproduced with permission.^[^ ^142^ ^]^ Copyright 2024, John Wiley and Sons. Additionally, it presents the in situ absorption spectra of both the (h) control and target CsPbI_2_Br films with their aging time. (i) Cross‐sectional SEM image of CsPbBr_3_ PSC and chemical structure of the ionic liquid DTPT. Reproduced under the terms of Creative Commons Attribution‐NonCommercial 3.0 Unported License.^[^ ^270^ ^]^ Copyright 2023, Royal Society of Chemistry.

Most studies on inorganic PSCs have considered an n–i–p structure. In 2014, Choi et al. reported the doping of Cs^+^ in MAPbI_3_ to improve the stability of PSCs. Although the focus was not on developing inorganic perovskites, MA^+^ was replaced entirely with Cs^+^ in MAPbI_3_ to form CsPbI_3_ as part of this study.^[^ ^14^ ^]^ The team obtained a negligible efficiency of 0.09% for CsPbI_3_. A breakthrough was achieved the following year when Eperon et al. stabilized the black phase of CsPbI_3_ at 100 °C with the addition of hydroiodic acid (HI) and fabricated CsPbI_3_ perovskite‐based solar cells to obtain an efficiency of 2.9% with a planar configuration in the device structure FTO|c‐TiO_2_|CsPbI_3_|spiro‐OMeTAD|Au.^[^ ^15^ ^]^ Thereafter, researchers have significantly focused on enhancing the performance of inorganic PSCs, leading to significant progress in recent years (Figures 1a and 8a). Notably, Wang et al. and Xu et al. substantially progressed in enhancing the efficiency of n–i–p‐type devices by utilizing interface engineering to stabilize the black phase (γ) of CsPbI_3_ by applying 4‐fluorobenzothiohydrazide (FBTH) as a perovskite precursor additive and 4‐amino‐2,3,5,6‐tetrafluorobenzoate cesium at the buried interface between TiO_2_ and perovskite, respectively, for defect passivation. PCEs of 21.15% and 21.41% were achieved with precursor additive and buried interface modification, respectively.^[^ ^19^, ^245^, ^246^ ^]^ A significant improvement was observed compared to their respective control device PCEs of 19.07% and 19.30%. Yang et al. employed ammonium formate molten salt as a precursor additive to control the crystallization leading to a record efficiency of 21.85% for γ‐CsPbI_3_.^[^ ^247^ ^]^ Zhao and co‐workers deposited a thin layer of zwitterion salt (cesium (2S,3S)‐3‐amino‐2‐methyl‐4‐oxoazetidine‐1‐sulfonate, MOCs) on TiO_2_ and achieved PCE of 20.67% from α‐CsPbI_3_.^[^ ^247^ ^]^ Qiu et al. further improved the performance by creating a dipolar chemical bridge (DCB) between the TiO_2_ ETL and perovskite using 3‐amino‐1‐propanesulfonic acid molecules (**Figure** **8**b,c) and suppressing the interfacial energy loss, thereby achieving a record performance of 21.86% with *V*_oc_ of 1.26 V.^[^ ^248^ ^]^ It is important to note that almost all of the passivation or additive engineering strategies utilize the DMAPbI_3_‐assisted precursor route for stabilizing the black CsPbI_3_ perovskite phase and addressing bulk and surface defects.^[^ ^19^, ^22^, ^45^, ^237^, ^249^, ^250^, ^251^, ^252^, ^253^, ^254^ ^]^ Recently, Mali et al. explored the construction of γ‐ and β‐phase heterojunctions of CsPbI_3_ and reported record performances of 21.59%.^[^ ^22^ ^]^ To minimize the negative impact of polar solvents on the underlying layer, the researchers developed a constructive approach by combining the solution process with evaporation to fabricate a β‐ and γ‐phase interface (Figure 8f). In the study, the one‐step dynamic hot‐casting technique, aided by DMAI and Zn(C_6_F_5_)_2_, enabled obtaining β‐CsPbI_3_ on top of a c‐TiO_2_/SnO_2_ bilayer ETL. The GAI‐assisted evaporation technique proved effective in developing γ‐CsPbI_3_ thin films.

Liu et al. fabricated CsPbBr*_x_* I_3−_ *_x_*‐based solar cells with an efficiency of 21.8% by employing Boc‐S‐4‐methoxy‐benzyl‐l‐cysteine as a passivator on perovskite films to suppress halide vacancies and coordinate with undercoordinated Pb^2+^.^[^ ^21^, ^255^, ^256^ ^]^ Wang et al. focused on modulating crystallization dynamics by extended annealing in air and incorporating 2,6‐pyridine carboxamide on top of perovskite films to passivate uncoordinated Pb^2+^, thereby suppressing defect‐induced recombination at an interface.^[^ ^18^ ^]^ The small‐molecule passivation strategy helped reduce *V*_oc_ loss significantly, providing a *V*_oc_ of 1.34 V and a record efficiency of 22.07%. Li et al. established that adding a methylamine acetate ionic liquid to the precursors boosted the formation of intermediate phases. Thus, the direct crystallization of CsPbI_2_Br due to ionic precursors was avoided. Furthermore, the method enabled the creation of a pure‐phase CsPbI_2_Br film under mild conditions. When combined with dopant‐free and stable P3HT, solar cells achieved an efficiency of 18.14%, setting a new record for CsPbI_2_Br‐solar cells (Figure 8g,h).^[^ ^142^ ^]^

In the case of CsPbIBr_2_, some researchers employed a triple‐component precursor strategy to overcome the solubility issues of Br precursor components. Thus, films of 390 nm thickness using the precursor solution that partially substituted PbBr_2_ with PbI_2_ in DMSO enhanced light absorption and resulted in a remarkable efficiency of 12.8%.^[^ ^257^ ^]^

Inorganic PSC research is increasingly being conducted on the p–i–n structure because of its promising stability and compatibility with the fabrication of perovskite–Si tandem solar cells, for which CsPbI_3_ provides an optimal bandgap (≈1.7 eV) that complements Si.^[^ ^124^, ^125^, ^126^, ^127^ ^]^ Li et al. introduced multifunctional ethacridine lactate (EAL) as an additive in the precursor solution to mitigate moisture ingress and suppress nondesirable defects, leading to better charge transport and efficiency of 21.08% with the glass/ITO/PEDOT:PSS/Me‐4PACz/CsPbI_3_:EAL/PCBM/C60/BCP/Ag architecture.^[^ ^161^ ^]^ Incorporating Me‐4PACz as a functional additive in the precursor and EAL improved the efficiency to 21.38%.^[^ ^162^ ^]^ Simultaneously, interface engineering in mixed anion inorganic CsPbI_3−_ *_x_* Br*_x_* films with 2‐mercapto‐1‐methylimidazole (MMI) enabled an efficiency of 20.6%.^[^ ^258^ ^]^ The results suggested that the mercapto group preferentially reacted with the undercoordinated Pb^2+^ in the perovskite, forming Pb─S bonds, thereby reducing the surface trap density. Furthermore, the MMI modification facilitated improved energy‐level alignment with the electron‐transporting material, fostering smoother carrier transfer and minimizing voltage deficits. Recently, the authors re‐established their study using another cross‐linker, (3‐mercaptopropyl) trimethoxysilane, and obtained an improved performance of 21.0% with the same device architecture.^[^ ^259^ ^]^ Wang et al. used 3‐amino‐5‐bromopyridine‐2‐formamide (ABF) (Figure 8d,e) in CH₃OH for passivation and defect‐rich surface treatment, forming vertical PbI_2_ nanosheet structures. Thus, localized contacts were enabled between the CsPbI_2.85_Br_0.15_ film and PCBM, suppressing the recombination of electron–hole pairs and benefiting electron extraction, resulting in an efficiency of 20.8%.^[^ ^260^ ^]^

Despite recent advances, inverted (p–i–n) inorganic PSCs have relatively lower efficiency and more considerable voltage loss than n–i–p structures. Primarily, the lack of suitable materials for charge transport poses challenges. The materials required for growing inorganic perovskite films, such as p‐type metal oxides and polymeric HTL, often require high annealing temperatures and significant synthesis costs. Additionally, their hydrophobic nature presents challenges that limit their widespread application and commercialization. Another challenge for inorganic PSCs, particularly those containing Br‐rich CsPbBr_3_ and CsPbIBr_2_, is the need for a two‐step synthesis process owing to the low solubility of CsBr. The process can result in uncontrolled crystallization and the formation of unwanted secondary phases such as Pb‐rich (CsPb_2_Br_5_) and Cs‐rich (Cs_4_PbBr_6_) phases.^[^ ^192^, ^193^, ^261^ ^]^ The high cost of polymer HTLs such as PTAA and electrodes (Au) has raised concerns about their long‐term stability and cost‐effective commercialization. Consequently, researchers are exploring novel HTLs suitable for inorganic perovskites. Nickel oxide (NiO*_x_*) is characterized by favorable band alignment with inorganic perovskites, making it one of the most widely used inorganic hole transport materials to enhance the performance and stability of inorganic PSCs.^[^ ^262^, ^263^ ^]^ However, Xu et al. found that having an organic self‐assembled monolayer Br‐2PACz on top of NiO*_x_* nanocrystals could enhance the efficiency to 19.34% from 15.2% for CsPbI_3_ and demonstrate excellent thermal stability.^[^ ^264^ ^]^ In addition to NiO*_x_*, CuI and CuSCN were successfully incorporated to get over 15% PCE in CsPbI_2_Br. Tang et al. explored innovative inorganic hole transport layers, such as Cu(Cr, Ba)O_2_ nanocrystals, CuInS_2_/ZnS quantum dots, and brominated graphene oxide (Br‐GO), for use in CsPbBr_3_ perovskite solar cells.^[^ ^265^, ^266^ ^]^ These findings indicate that carefully engineered inorganic HTMs have significant potential for developing efficient and stable inorganic perovskite solar cells.

However, significant progress has been made in terms of commercialization prospects and performance. Expensive Au electrodes have been replaced with C‐counter electrodes in HTL‐free structures, which have the advantages of low‐cost fabrication and superior stability.^[^ ^267^ ^]^ Zhang et al. achieved an efficiency of 18.05% for C‐based HTL‐free inorganic PSCs (C‐PSCs) with a planar device architecture (FTO/c‐TiO_2_/CsPbI_3_/C).^[^ ^166^ ^]^ Recently, studies have further improved to record an efficiency of 18.34% using a more energy‐aligned 1D perovskite (5‐azaspiro[4.4]nonan‐5‐ium Pb triiodide, ASNPbI_3_) as a capping layer on top of the perovskite to suppress defects in HTL‐free C‐PSCs.^[^ ^268^ ^]^ Despite the exciting progress in the efficiency of inorganic PSCs, the reported efficiencies still fall short of the Schockley–Quiesser theoretical limit.^[^ ^269^ ^]^ For instance, CsPbBr_3_, with its wide bandgap of ≈2.3 eV, has a theoretical PCE limit of 16.5%, of which 11.21% has been realized using ionic liquid (dimethylamino)‐1‐(2,2,2‐trifluoromethyl) pyridine‐1‐mum 2,2,2‐trifluoroacetate (DTPT) (Figure 6i) in between interfacial contacts.^[^ ^37^, ^270^ ^]^ Efficiency disparity can be attributed to the more complex formation, phase transformation, and crystallization processes of CsPbBr_3_.^[^ ^146^, ^249^ ^]^

In addition, their wider bandgap compared to hybrid perovskites has always captured the interest of researchers in tandem solar cell development and led to comprehensive SCAPS‐ID simulations.^[^ ^271^, ^272^, ^273^, ^274^ ^]^ However, challenges such as phase instability and processing constraints have limited their practicality. Recent works showcased the potential of CsPbI*_x_* Br_3−_ *_x_* when paired with crystalline silicon two‐terminal (2‐T) tandems, achieving a PCE of 22.95% while maintaining 95.7% of their performance after 300 h of illumination.^[^ ^275^ ^]^ They further enhanced this to 25.31% by incorporating 2‐amino‐5‐bromobenzamide (ABA) atop CsPbI_2.85_Br_0.15_.^[^ ^276^ ^]^ Additionally, inorganic–hybrid (CsPbI_3−_ *_x_* Br*_x_*/FA_0.7_MA_0.3_Pb_0.5_Sn_0.5_I_3_) 2‐T achieved a PCE of 25.6%,^[^ ^272^ ^]^ and fully inorganic perovskite‐based (CsPb_0.4_Sn_0.6_I_3_/CsPbI_2_Br) reached a PCE of 22.57%^[^ ^277^ ^]^ highlighting a promising trajectory for inorganic‐based tandem solar cells.

It is crucial to prioritize the operational stability and efficiency of the device together, as this directly impacts the energy payback time for the PSC technology. Despite the black phase's tendency to spontaneously transform into a nonphotoactive phase, especially under humid conditions due to its thermodynamic instability at room temperature,^[^ ^225^, ^278^, ^279^ ^]^ efforts have been focused on minimizing the influence of external factors. Consequently, several cutting‐edge devices have achieved stability for over 1000 h, retaining over 90% of the initial PCE under white light LED (w‐LEDs) in an inert environment.^[^ ^20^, ^41^, ^122^, ^155^, ^159^, ^244^, ^266^, ^268^, ^278^, ^280^, ^281^, ^282^, ^283^, ^284^, ^285^, ^286^, ^287^, ^288^ ^]^ Additionally, a few studies have demonstrated a *T*_80_ exceeding 1000 h for unencapsulated devices illuminated under ambient conditions with a relative humidity above 20%. Section 7 provides a comprehensive overview of the latest developments in device operational stability.

## 6 Large‐Area Inorganic Solar Cells

**Figure 9:**
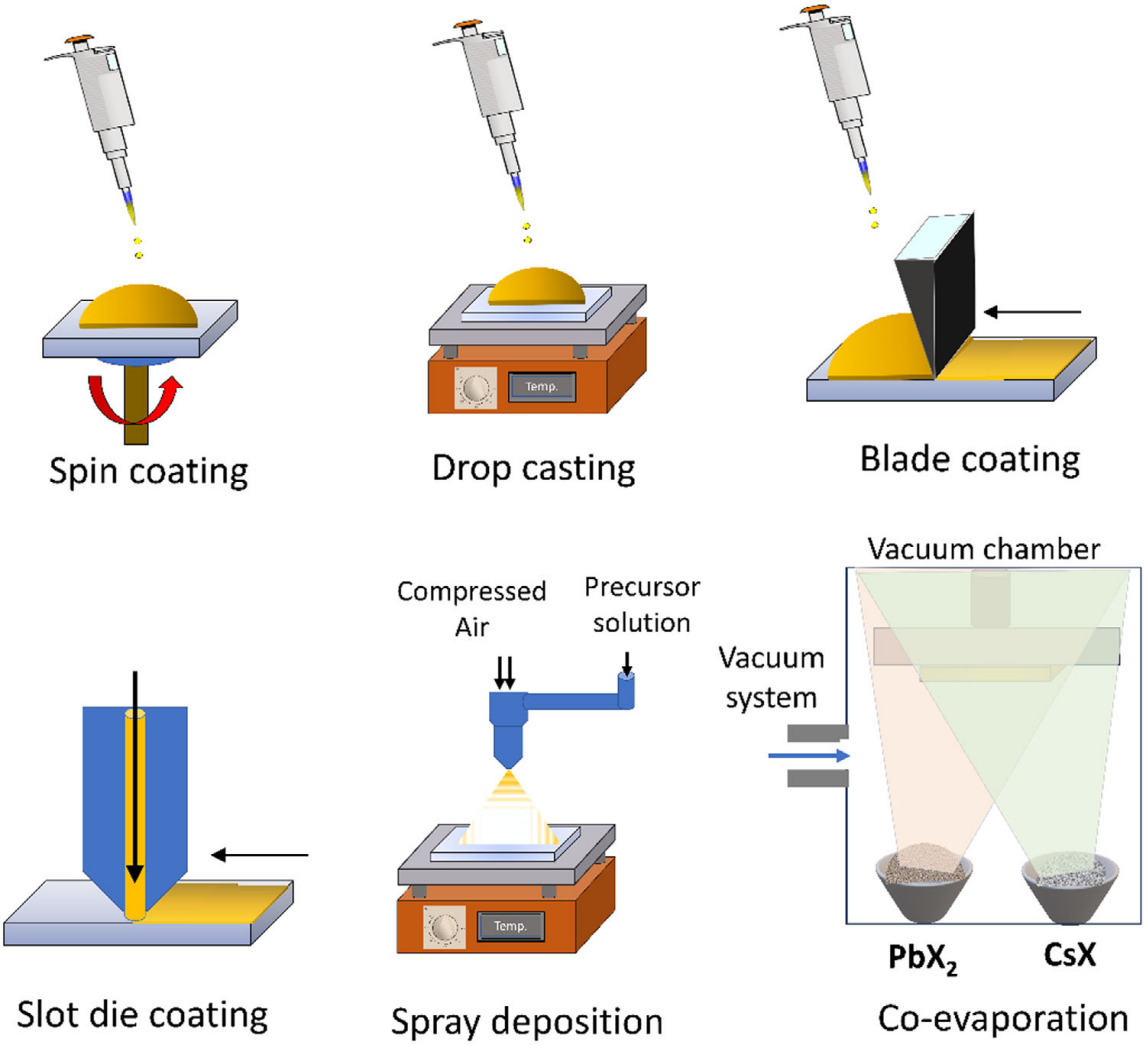
Schematic of deposition methods used for the large area (≥1 cm^2^) inorganic devices.

Research on PSCs has recently made significant strides in demonstrating the adaptability of lab‐scale processes to large‐area applications. Although hybrid PSCs have showcased large‐area modules exceeding 200 cm^2^ with an efficiency of 20.6%,^[^ ^289^ ^]^ inorganic PSCs currently lagging in large‐area applications. A record lab‐scale efficiency of over 22% in inorganic solar cells has sparked enthusiasm among researchers to prioritize scalability and manufacturability over larger areas through innovative fabrication methods. **Figure** **9** and **Table** **2** present a diverse range of reported techniques for fabricating large‐area inorganic PSCs. The methods encompass various solution‐based vapor‐deposition approaches such as spin coating, dip coating, doctor blading, slot die coating, inkjet printing, spray coating, vacuum flash‐assisted solution, and chemical vapor deposition. Physical vapor‐assisted evaporation methods have been explored, including sequential and coevaporation and combinations of solution and evaporation methods. An active area of 1 cm^2^ is commonly considered the threshold representation for a large area, representing a substantial increase over a typical lab‐scale device area of generally ≤10 mm^2^. Expanding beyond the threshold is achievable without a significant loss in sheet resistance and geometrical fill factor (GFF) through the interconnection of stripes that are generally 1 cm wide. GFF is crucial for PV modules because it signifies the ratio of the aperture area to the total area of the module. Higher GFF in high‐performance inorganic PSCs increases energy generation within a given module size, underscoring the significance of the field.

**Table 2:** Deposition methods, strategies, architecture, and corresponding performance of large‐area (≥1 cm^2^) devices.

Perovskite	Method	Additive/strategy	Phase	Deposition envt.	Annealing temp. [°C], time	Annealing envt.	Architecture	Active area [cm^2^]	*V*_oc_ [V]	*J*_sc_ [mA cm^−2^]	FF [%]	PCE [%]	Refs.
CsPbI_3_	Spin coating	DMAI, [PPN][TFSI]	γ	Inert	100, 10 180, 20	Air	FTO/c‐TiO_2_/SnO_2_/CsPbI_3_:[PPN][TFSI]/spiro‐OMeTAD/Au	28.17^b)^	9.60	2.51	67.4	16.9	[305]
	Spin coating	DMAI; CHI post‐treatment	β	Inert	210, 5	Drybox (<10% RH)	FTO/c‐TiO_2_/CsPbI_3_:CHI/spiro‐OMeTAD/Ag	1.0	1.11	19.6	74.0	16.1	[291]
	Spin coating	PEAI passivation, vacuum‐assisted thermal annealing	γ	Inert	200, 2	Vacuum	FTO/TiO_2_/CsPbI_3_/PEAI/spiro‐MeOTAD/Au	1.0	1.12	20.2	74.3	16.8	[293]
	Spin coating Blade coating	18‐crown‐6 ether (crown)crystal passivation, PbI_2_·NMP additive, HI	α	Inert	170, 30	Inert	FTO/ZnO–ZnS/m‐TiO_2_/CsPbI_3_–CsPbI_3_‐crown/spiro‐OMeTAD/Au	8.0 ^b)^ 8.0 ^b)^	4.05 3.84	4.10 4.05	71.5 69.0	11.9 10.7	[292]
	Blade coating	DMAPbI3	γ	Inert	70, 2–5 190, 10	Inert Air	FTO/c‐TiO_2_/CsPbI_3_:BTABr/spiro‐OMeTAD/Au	12	5.56	19.4	77.3	16.6	[34]
	Blade coating	DMAI, Zn(C_6_F_5_)_2_ Phase heterojunction GAI‐assisted coevaporation	β γ	Ambient, 25–30 RH%	60, 5 100, 10 210, 20 100, 3	Ambient, 25–30 RH%	FTO/c‐TiO_2_/SnO_2_/β‐γ‐CsPbI_3_/PC_61_BM/P3HT‐SMe‐TATPyr/Ag	18.08 ^b)^	7.30	345	73.2	18.4	[67]
	Coevaporation												
	Ultrasonic spraying	MXene, DMAI	γ	20	170, 20	20	FTO/PTAA/CsPbI_3_/OMXene‐CsPbI_3_/CPTA/BCP/Ag	25 ^b)^	3.57	143	71.6	14.6	[148]
CsPbI_3−_ *_x_* Br*_x_*	Spin coating	Boc‐S‐4‐methoxy‐benzyl‐L‐cysteine (BMBC) passivation, HPbI_3_	γ	Inert	210, 3	Inert	FTO/c‐TiO_2_/CsPbI_2.85_Br_0.15_/BMBC/spiro‐OMeTAD/Au	1.0	1.19	20.6	70.0	17.2	[306]
	Spin coating	2,6‐Diaminopyridine (2,6‐DAPy) passivation, HPbI_3_	γ	Inert	210, 5	Inert	FTO/c‐TiO_2_/CsPbI_2.85_Br_0.15_/2,6‐DAPy/spiro‐OMeTAD/Au	1.0	1.21	20.2	70.2	17.1	[44]
	Spin coating	2‐Mercapto‐1‐methylimidazole (MMI) passivation, HPbI_3_	γ	Inert	210, 5	Inert	FTO/NiO*_x_*/CsPbI_2.8_Br_0.2_/MMI/PCBM/BCP/Ag	1.0	1.19	20.1	72.7	17.3	[51]
	Spin coating	HPbI_3_, TFA passivation	γ	Inert	210, 5	15–25% RH	FTO/c‐TiO_2_/CsPbI_2.85_Br_0.15_:TFA/spiro‐OMeTAD/Au	1.0	1.20	20.6	70.0	17.2	[294]
	Spin coating	HPbI_3_	α	Inert	N.M, 25	Inert	FTO/TiO_2_/CsPbI_2_._85_Br_0.15_/PTAA/Au	1.0	1.04	19.0	61.9	12.3	[307]
	Blade coating	2D–3D Cs_2_PbI_2_Cl_2_–CsPbI_2.5_Br_0.5_ interface	α	Inert	325, 10	Inert	FTO/c‐TiO_2_/Cs_2_PbI_2_Cl_2_–CsPbI_2.5_Br_0.5_/spiro‐OMeTAD/MoO_3_/Ag	1.0	1.09	17.8	65.8	12.7	[308]
								2.0	1.12	16.2	55.3	10.0	
	Ultrasonic spray coating	Sequential spray of CsPbI_3_ onto CsPbI_2_Br film		Inert	150, N.M	≈20%	FTO/c‐TiO_2_/CsPbI_3−_ *_x_* Br*_x_*/PTAA/Au	112.0	7.64	281	72.1	13.8	[309]
CsPbI_2_Br	Spin coating	InCl_3_, TPFPB, and LiClO_4_ doped C_60_; polyimide tape spacer‐assisted annealing (quasi‐curved heating)	α	Inert	42 160 °C with PI tape 160, 10	Inert	FTO/NiO/CsPbI_2_Br/ZnO/C60 + TPFPB + LiClO_4_/Ag	1.0	1.25	15.8	73.0	14.4	[298]
								10.92 ^b)^	4.56	41.3	71.0	12.2	
	Spin coating	InCl_3_ doping; polyimide tape spacer‐assisted annealing		Inert	42 160 °C with PI tape 160, 10	Inert	FTO/NiO*_x_*/InCl_3_:CsPbI_2_Br/ZnO@C_60_/Ag	1.0	1.10	15.5	67.0	11.4	[297]
	Spin coating	BaI_2_‐additive, dynamic hot‐air (100 °C) blowing	α	Inert	280, 10	Ambient	FTO/c‐TiO_2_/CsPbI_2_Br/P3HT/Au	1.0	1.22	15.3	74.3	13.8	[124]
	Spin coating	Methylamine (MA) gas healing method		N.M	100, 2	N.M	FTO/c‐TiO_2_/CsPbI_2_Br⋅0.5MAI_0.3_Br_0.7_/spiro‐OMeTAD/Ag	1.0	1.10	14.5	63.0	10.0	[310]
	Spin coating	NiO*_x_* as HTL and Nb_2_O_5_ as ETL		Inert	42, 4 160 °C with PI tape 160, 10	Inert	ITO/NiO*_x_*/CsPbI_2_Br/Nb_2_O_5_/Ag	1.0	1.17	13.8	76.3	12.3	[299]
								5.0	1.14	13.5	72.5	11.2	
	Spin coating	Addition of PbI_2_(DMSO)_2_ and PbBr_2_(DMSO)_2_		Inert	200, 10	Inert	FTO/TiO_2_/CsPbI_2_Br/spiro‐OMeTAD/Au	1.0	1.16	14.7	68.0	11.6	[296]
	Blade coating	PbI_2_(DMSO)_2_ and PbBr_2_(DMSO)_2_		30–50%	150, 10	30–40%	FTO/TiO_2_/CsPbI_2_Br/spiro‐OMeTAD/Au	1.0	1.18	15.3	69.0	12.5	[203]
	Ultrasonic spray coating	Orthogonal spray‐coating		Inert	150, N.M	≈20%	FTO/c‐TiO_2_/CsPbI_2_Br/PTAA/Au	1.0	1.22	14.8	76.7	13.9	[309]
								24.0 ^b)^	1.20	14.0	75.4	12.7	
								48.0 ^b)^	1.17	13.9	74.0	12.0	
								80.0 ^b)^	1.13	14.0	71.0	11.2	
								112.0 ^b)^	1.09	14.2	69.6	10.8	
	Spin coating			N.M	N.M	N.M		1.0	1.20	14.6	78.9	13.8	
								24.0 ^b)^	1.18	13.8	71.2	11.5	
								48.0 ^b)^	1.10	13.7	66.5	10.0	
								80.0 ^b)^	1.01	13.1	58.8	7.78	
								112.0 ^b)^	0.91	12.7	51.5	5.96	
	Coevaporation	Dual‐source evaporation	α	Vacuum	350, 10	Inert	FTO/c‐TiO_2_/CsPbI_2_Br/P3HT/Au	1.2	1.02	11.5	58.0	6.80	[311]
CsPbBr_3_	Spin coating Followed by dip coating	Cu‐phthalocyanine (CuPc) as HTL		Inert	80, 30 250, 5	Inert	FTO/c‐TiO_2_/m‐TiO_2_/CsPbBr_3_/CuPc/carbon	2.25	1.28	5.69	64.5	4.72	[301]
	Spin coating Followed by dip coating	SnO_2_/TiO_2_ bilayer with CuPc HTL		Inert	90, 60 250, 5	Inert	FTO/SnO_2_/c‐TiO_2_/m‐TiO_2_/CsPbBr_3_/CuPc/carbon	1.0	1.39	6.93	71.0	6.90	[97]
	Spray‐assisted deposition	MoS_2_ QDs HTL Spray‐coating CsBr on top of spin‐coated PbBr_2_		Ambient	80, 30 250, 10	Ambient	FTO/c‐TiO_2_/m‐TiO_2_/CsPbBr_3_/MoS_2_/carbon	1.0	1.24	4.93	67.2	4.12	[127]
								1.5	1.23	4.47	64.2	3.54	
								2.0	1.21	4.33	60.4	3.15	
								3.0	1.13	4.14	49.7	2.33	
	Co‐evaporation	Dual‐source evaporation and annealing	α	Vacuum	300, N.M	Inert	FTO/c‐TiO_2_/CsPbBr_3_/spiro‐MeOTAD/Au	1.0	1.32	5.11	79.3	5.37	[129]

^a)^ Not mentioned in the literature;

^b)^ Submodules; Inert ‐ Glovebox; QD ‐ Quantum dot.

Solution‐based spin‐coating method plays a pivotal role in advancing inorganic PSCs research. However, spin coating is not a scalable technique for large‐scale industrial manufacturing of solar cell modules. Nevertheless, numerous attempts have been made to achieve a large area (≥1 cm^2^) of inorganic PSCs via spin coating. Jiang et al. fabricated mirror‐like CsPbI_3_ (9 × 9 cm^2^) by precursor engineering using CsAc and HPbI_3_. Additionally, they incorporated phenethylammonium iodide (PEAI) into the precursor, which induced phase‐stable quasi‐2D mixed α‐CsPbI_3_ formation.^[^ ^290^ ^]^ Although an efficiency of 12.4% was reported with single‐step spin coating for an active area of 0.16 cm^2^, the performance of large‐area films was not mentioned. Further, Wang et al. thermodynamically stabilized β‐CsPbI_3_ using HPbI_3_ and interface engineered with passivator choline iodine (CHI) to obtain an efficiency of 16.1% from 1 cm^2^ devices.^[^ ^291^ ^]^ To address the challenges of moisture‐induced phase transition and defect migration in CsPbI_3_, Chen et al. used 18‐crown‐6 ether (crown) as a passivator.^[^ ^292^ ^]^ The outer structure of the crown, particularly the ─CH_2_ group, offered adequate protection against moisture, while the inner cavity passivated surface defects by forming robust bonds with the surface Cs ions. Through innovative use of organic molecules, they achieved efficiencies of 16.9% and 11.8% for small‐area (0.1 cm^2^) and large‐area (4 × 4 cm^2^ module with 8 cm^2^ active area) inorganic (CsPbI_3_) solar cells, respectively. Yu et al. employed vacuum‐assisted thermal annealing to control the morphology and crystallinity of CsPbI_3_ perovskite films fabricated by spin coating. They achieved a PCE of 16.8% for a 1 cm^2^ device.^[^ ^293^ ^]^

Despite their known thermodynamic instability, Li et al. strategically employed a 2D inorganic PSC modification for large‐area devices. They synthesized 2D CsPbI_2_Cl_2_, which is a more stable alternative to other 2D inorganic PSCs. By utilizing a 2D–3D (CsPbI_2_Cl_2_‐CsPbI_2.5_Br_0.5_) heterostructure, they achieved PCEs of 12.74% and 10.01% for 1 and 2 cm^2^ devices, respectively. Incorporating Cs_2_PbI_2_Cl_2_ promoted the (100) preferred crystal orientation of CsPbI_2.5_Br_0.5_, and significantly enhanced the crystal quality, uniformity, and repeatability, resulting in improved carrier transport and performance. Zhang et al. employed trifluoroacetamidine (TFA) to suppress CsPbI_2.85_Br_0.15_ film defects, yielding a PCE of 17.21% from a 1 cm^2^ device area.^[^ ^294^ ^]^ Saliba and Abate optimized the I/Br ratio for a phase‐stable scalable formulation using pulsed flash infrared annealing (p‐FIRA)‐assisted spin coating.^[^ ^295^ ^]^ They found that a 60%/40% I/Br ratio (CsPbI_1.8_Br_1.2_) was ideal for achieving phase stability without compromising desired absorption.

Given the relatively favorable phase stability and the synthetic advantage of the solvable solubility of CsBr and PbI_2_, CsPbI_2_Br has garnered more global attention than any other inorganic perovskite formulation for scaling up. Additionally, the utilization of DMSO adducts (PbI_2_·DMSO and PbBr_2_·DMSO) significantly enhances the grain size of spin‐coated CsPbI_2_Br films, resulting in low defect densities, prolonged carrier lifetimes, and high stability.^[^ ^296^ ^]^ The prepared CsPbI_2_Br films demonstrate a high open‐circuit voltage of 1.16 V and a PCE of ≈11.61% for 1 cm^2^ devices. Liu et al. attained over 11% efficiency for 1 cm^2^ inverted (FTO/NiO_x_/CsPbI_2_Br/ZnO@C_60_/Ag) devices by structurally reconstructing CsPbI_2_Br perovskite through In^3+^ and Cl^−^ codoping,^[^ ^297^ ^]^ which improved overall spatial symmetry with a closely packed atom arrangement due to the crystal structure transformation from orthorhombic (*Pnma*) to cubic (*Pm‐3m*). They employed spin coating, followed by a novel thermal radiation heating method, to achieve uniformity on a large scale. Subsequently, they introduced a nonhygroscopic lithium salt (LiClO_4_) to enhance the electron mobility and conductivity of the film. They refined the process by implementing quasi‐curved heating, in which the spin‐coated films were heated on a curved glass surface, resulting in efficiencies of 14.44% for a 1 cm^2^ device and 12% for a 10.92 cm^2^ module.^[^ ^298^ ^]^ NiO*_x_* ETL and Nb_2_O_5_ HTL produced using scalable e‐beam evaporation techniques were utilized along with spin‐coated CsPbI_2_Br to achieve efficiencies of 12.33% and 11.2% on 1 and 5 cm^2^ devices, respectively.^[^ ^299^ ^]^ Additionally, 13.78% efficiency (1 cm^2^) was achieved by the dynamic hot‐air‐assisted spin coating of BaI_2_‐added CsPbI_2_Br films.^[^ ^124^ ^]^

The fabrication of large‐area CsPbBr_3_ solar cells has shown significant progress with multistep spin coating, similar to their small‐area counterparts. Li et al. achieved an efficiency of 7.07% for 1 cm^2^ Cs_0.91_Rb_0.09_PbBr_3_‐based device constructed via two‐step spin coating.^[^ ^300^ ^]^ Liu et al. spin‐coated a PbBr_2_ layer on a TiO_2_/FTO substrate, followed by multiple dip‐coatings in a CsBr–methanol solution at 55 °C to produce a CsPbBr_3_ film. The resulting CsPbBr_3_ film, in combination with CuPc/C for an active area of 2.25 cm^2^, provided a PCE of 4.72%.^[^ ^301^ ^]^ Further, the method was modified on a SnO_2_/TiO_2_ bilayer to achieve a PCE of 6.9% for a 1 cm^2^ device.^[^ ^97^ ^]^

Drop casting is an intriguing method owing to its simplicity, scalability, low‐waste nature, and compatibility with large‐scale production. The process involves depositing materials from a solution by simply dropping them onto a substrate, making it an attractive option for numerous applications. However, an unavoidable issue known as the coffee ring effect can occur, leading to an inhomogeneous distribution of nonvolatile solutes and nonuniform film fabrication owing to faster solvent evaporation at the edge region. Zhang et al. addressed the problem by utilizing a vacuum‐assisted process after drop casting, allowing for conformal and accelerated solvent evaporation and yielding uniform and compact CsPbI_3_ perovskite films.^[^ ^302^ ^]^ Their technique enabled the creation of uniform 5 × 5 cm^2^ films that were further processed into 1 cm^2^ devices with an active area of 0.0625 cm^2^, achieving a PCE of 16%. Although the process was easily scalable, they recommended using dry air or a <20% RH environment to avoid humidity‐induced degradation. Xiao et al. addressed the processing condition challenges by combining airflow drying and PbAc_2_·3H_2_O additive to produce uniform CsPbI_2.25_Br_0.75_ films in humidity up to 50% RH.^[^ ^303^ ^]^ The perovskite films prepared using the additive‐assisted airflow drying (AAD) method exhibited larger grains, higher crystallinity, and fewer defects than the control films. Solar cells with CsPbI_2.25_Br_0.75_ prepared using the AAD method achieved a PCE of 15.22% from 1 cm^2^ active area devices.

Blade coating is another scalable fabrication technique extensively studied for inorganic PSCs, specifically for CsPbI_3_ films. Chen et al. used a crown for the passivation of perovskites, which led to the exploration of blade coating as an alternative to spin coating for the fabrication of mini CsPbI_3_ modules.^[^ ^292^ ^]^ A 4 µL solution of CsPbI_3_ was dispensed into the 200 µm gap between the blade and the substrate and then moved at a controlled speed of 200 mm s^−1^. The process was followed by a 1 min vacuum flash and subsequent annealing in a customized glovebox with 10–15% RH. While spin coating resulted in a PCE of 11.87% for an 8 cm^2^ active area, blade coating yielded a similar PCE of 10.73%. A major reason for selecting vacuum flashing and processing under controlled environmental conditions after blade coating was the fast drying of the solution with uncontrolled airflow, resulting in low‐quality films. Researchers have used various precursor additives or passivation, such as EMIMHSO_4_,^[^ ^179^ ^]^ benzyltrimethylammonium bromide (BTABr),^[^ ^304^ ^]^ and Zn(C_6_F_5_)_2_,^[^ ^243^ ^]^ to precisely control the growth of perovskite films, defect passivation, and moisture‐induced phase instability under ambient conditions with regulated airflow during blade coating. While the ionic liquid EMIMHSO4‐based devices resulted in a PCE of 14.6% with a 1 cm^2^ device, passivation with quaternary bromide salt (BTABr) led to the creation of interfacial gradient heterostructures (referred to as BTA^+^–CsPbI_3−_ *_x_* Br*_x_*) at low temperatures, resulting in a PCE of 16.6% for a 12 cm^2^ minimodule. Recently, Mali et al. modified the additive‐assisted blade coating method with hot‐air flow while blade coating to develop a phase‐heterojunction device with β‐CsPbI_3_ and γ‐CsPbI_3_ (produced via GAI‐assisted triple‐source thermal evaporation).^[^ ^22^ ^]^ The amalgamation of two different methods exhibited a PCE of 19.58% for a 1 cm^2^ device and 18.43% for 18.08 cm^2^ active area modules (with a 13 × 13 cm^2^ module), implying a negligible loss of GFF (≈5.8%).

Currently, reports on the scalable fabrication of mixed‐halide inorganic PSCs are limited, providing an opportunity for further exploration in this domain. Fan et al. demonstrated the potential of blade coating CsPbI_2_Br with a DMSO adduct precursor, achieving a PCE of 12.5% for a 1 cm^2^ device.^[^ ^203^ ^]^ The study highlighted the potential for overcoming challenges related to moisture and following the Bénard‐Marangoni convection by optimizing the blade coating temperature from 80 to 150 °C for producing uniform and pinhole‐free CsPbI_2_Br films through sequential crystallization with adjustments in the halide composition of the intermediate film.

Although blade coating is widely used, it poses several limitations. The method is mainly suited for batch‐to‐batch processes such as on glass and does not offer freedom‐of‐design patterning. A substrate technique such as laser or mechanical scribing is required to fabricate blade‐coated films for large‐area module fabrication. On the contrary, slot die coating is a premetered technique that allows 1D patterning freedom in terms of coating stripes with high precision. The method is a continuous technique suited for batch‐to‐batch and high‐throughput roll‐to‐roll processes.^[^ ^312^ ^]^

Additionally, toxic solvents such as DMF and NMP, which have significantly advanced spin coating and blade coating, are not ideal for scalability. Considering these factors, Abate et al. introduced an environmentally friendly, solvent‐based, inorganic perovskite ink. The ink used noncoordinating, high‐vapor‐pressure solvents such as acetonitrile (ACN), 2‐methoxy ethanol (2‐ME), and the primary solvent DMSO, either individually or in combination, to produce large‐area CsPbI_2.77_Br_0.23_ films on preheated (40–50 °C) glass/FTO/c‐TiO_2_ substrates using a slot die coater in <20% RH, without requiring N_2_ knife blowing. The resulting films were annealed at 210 °C for 5 min.^[^ ^128^ ^]^The fabricated solar module with six subcells and an area of 10 × 10 cm^2^, using a DMSO and ACN solvent mix (0.8:0.2), achieved a PCE of 8.07%. A device with an active area of 2.5 × 2 cm^2^ obtained a PCE of 16.03%.

Zhang et al. employed a two‐step droplet inkjet printing process to produce phase‐pure CsPbBr_3_ perovskite films. They discovered that the quality of the intermediate phase, known as CsPb_2_Br_5,_ was linked to the purity and crystal structure of the CsPbBr_3_ film. The intermediate phase played a crucial role in the inkjet printing process, influencing the final quality and efficiency of the perovskite film.^[^ ^313^ ^]^ Their work resulted in the development of a 1 cm^2^ HTL‐free carbon‐based CsPbBr_3_ solar cell with an efficiency of 7.81%. Recently, Li et al. demonstrated the possibility of inkjet printing of CsPbBr_3_ nanocrystals on soft PVP substrates and observed that 80% of the fluorescence was retained after 210 d of ambient storage.^[^ ^314^ ^]^

Spray deposition—well‐suited for manufacturing processes involving low temperatures, low concentrations, and large areas—is another practical and adaptable method. Heo et al. advanced surface engineering by creating a composite layer of OMXene–CsPbI_3_ using a sequential orthogonal spray‐coating technique with CsPbI_3_ and CsPbI_3_/OMXene precursor solutions.^[^ ^148^ ^]^ They developed minimodules with active areas of 25 cm^2^, which exhibited a PCE of 14.64%. Heo et al. introduced an orthogonal spray‐coating technique for producing graded, inorganic CsPbI_3−_ *_x_* Br*_x_* perovskite thin films with improved processability. By spray‐coating the CsPbI_3_ precursor solution onto an existing CsPbI_2_Br perovskite film at 150 °C with N_2_ flow gas, they fabricated a well‐defined, graded CsPbI_3−_ *_x_* Br*_x_* in the surface region. The approach enabled the fabrication of graded CsPbI_3−_ *_x_* Br*_x_* PSC submodules via scalable spray‐coating. PCE decreased from 14.04% at 0.096 cm^2^ to 10.82% at 112 cm^2^ for the spray‐coated devices, suggesting uniformity challenges over large areas with the technique. Moreover, the PCE decreased significantly from 14.10% at 0.096 cm^2^ to 5.96% at 112 cm^2^ for the spin‐coated devices. Thus, a significant loss in voltage and fill factor due to film nonuniformity was observed owing to the changes in crystallization dynamics from the center to the edge of the film. Another approach involves investigating spray depositing CsX on top of spin‐coated PbX_2_ to achieve CsPbI_3−_ *_x_* Br*_x_* large‐area films.^[^ ^126^, ^127^ ^]^ Using the approach, CsPbBr_3_ devices with a 1 cm^2^ active area have been developed to exhibit an efficiency of 4.17%.^[^ ^127^ ^]^

However, the solubility limitations of CsBr and PbBr_2_ in commonly used solvents raise concerns about the reproducibility of stoichiometrically balanced CsPbBr_3_ films. Conversely, physical and chemical evaporation techniques effectively address constraints related to solubility. Thermal evaporation is a facile method for creating inorganic perovskite thin films. Ma et al. investigated the impact of stoichiometry on the stability of CsPbI_2_Br thin films and devices produced through dual‐source thermal evaporation.^[^ ^315^ ^]^ Films were formed through the coevaporation of CsBr and PbI_2_, followed by a postannealing process at 300 °C under a N_2_ atmosphere. The CsPbI_2_Br cell with stoichiometric balance achieved a PCE of 6.8% for a 1.2 cm^2^ cell.

Lei et al. used a dual‐source coevaporation approach with CsBr and PbBr_2_ precursors to fabricate CsPbBr_3_ perovskite films. The stoichiometry of the deposited CsPbBr_3_ films was affected by the ratio of the evaporation rates of CsBr and PbBr_2_, and the crystallinity of the CsPbBr_3_ films was determined by both the substrate and post‐annealing temperatures. Notably, the resulting PCE for 1 cm^2^ devices reached 5.37%.

Evaporation processing generates high‐quality films, but the energy requirements and low throughput associated with vacuum processing hinder large‐area fabrication. Achieving stable black‐phase CsPbX_3_ in large‐area devices necessitates annealing active layers at high temperatures (>200 °C). The development of large‐area, fully inorganic perovskites is a relatively recent area of study, and their overall performance still requires improvement. The complexities associated with inorganic perovskites have made fabricating large‐area devices more challenging. Further exploration and in‐depth research on large‐area, fully inorganic PSCs are essential. Identifying a scalable deposition technique is a necessary first step, and addressing the efficiency loss resulting from the expanded active areas and interconnections between subcells is crucial. Overcoming such challenges requires prioritizing the development of superior materials with enhanced quality and stability, making them suitable for commercial applications.

## 7 Device Stability

The increasing interest in inorganic PSCs is fueled by their potential to offer enhanced stability compared with their organic–inorganic hybrid halide perovskite counterparts. Although the long‐term stability of hybrid devices is under investigation, significant progress has been made in addressing intrinsic stability issues. Early‐stage stability analyses of laboratory‐scale solar cells are crucial for advancing emerging photovoltaics and expediting the transition from laboratory to large‐scale industrial production. However, the lack of consistency in the stability testing procedures and reporting in publications makes comparing data and gaining a comprehensive understanding of the factors contributing to cell degradation difficult, thereby hindering the progress in stability. Accordingly, research communities have proposed six standardized protocols to address significant stress factors for stability, such as light, moisture, temperature, and electrical bias.^[^ ^316^ ^]^ The protocols include ISOS‐D, ISOS‐L, ISOS‐O, ISOS‐T, ISOS‐V, ISOS‐LT, and ISOS‐ST for dark storage and shelf life, light soaking, outdoor, thermal cycling, electrical bias, light humidity–thermal cycling, and solar thermal cycling testing, respectively. However, most literature still reports stability testing procedures inconsistent with the ISOS protocols.

**Figure 10:**
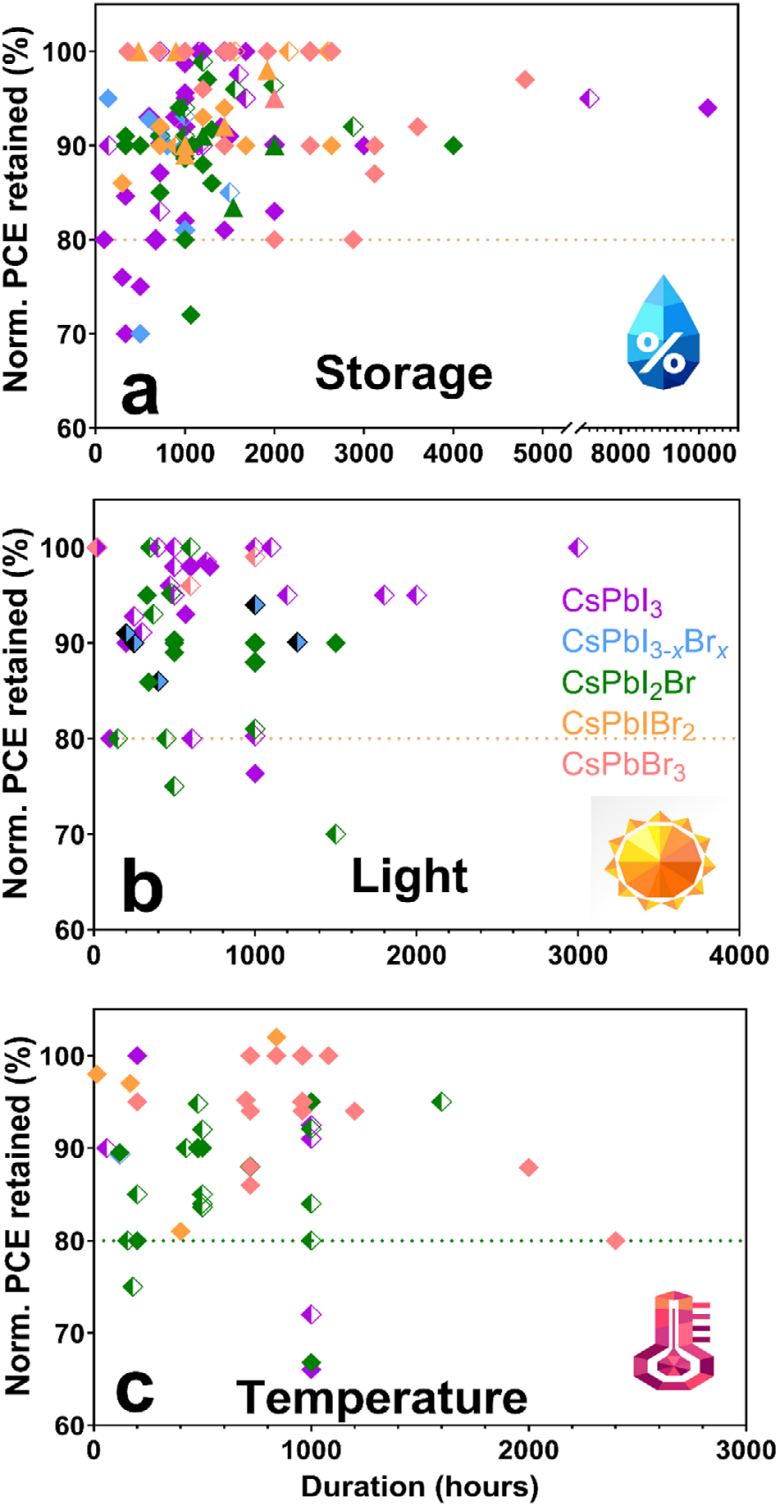
Long‐term stability of CsPbX_3_ devices in different external ingress conditions: a) dark storage, b) under illumination, and c) kept at 85 °C. Solid symbols represent unencapsulated devices, and half solid‐half white symbols represent encapsulated devices or devices measured under nitrogen.

Inorganic perovskite displays high thermal stability with the onset of degradation beyond 400 °C.^[^ ^41^ ^]^ Contrarily, the onset of thermal decomposition of organic–inorganic hybrid perovskites, including MAPbI_3_ and FAPbI_3_, occurs at temperatures as low as 60 and 80 °C, respectively.^[^ ^317^, ^318^ ^]^ While the thermal stability of inorganic perovskite is not the bottleneck in inorganic PSCs, the susceptibility of spontaneous phase transformation from a photoactive α‐phase into a nonphotoactive δ‐phase in the presence of moisture remains the most critical challenge during processing under an ambient environment during operational and storage lifetime (shown in **Figure** **10** and **Table** **3**).^[^ ^278^ ^]^ In CsPbI_3_ thin films, the α → δ phase transition is reported to occur at 23 °C and 11% RH atmosphere within 18 h, while the same samples sealed under N_2_ atmosphere remain stable in the α‐phase at 23 °C and withstand temperatures up to 200 °C for several hours.^[^ ^319^ ^]^ Several advances have been made to alleviate the susceptibility to phase transformations, widen the processing window, and improve the operational stability under humid conditions.

**Table 3:** Selected stability results of CsPbX_3_ devices in different analysis conditions.

Stability	PCE^a)^	Norm. PCE retained [%]	Duration [h]	Testing conditions (lamp power/temp./env./encap.)	Architecture	Refs.
Storage	20.98	91	1500	Xenon (100 mW cm^−2^)/20–30 °C/20–30% RH/unencap.	FTO/TiO_2_:3‐sulphonatopropyl acrylate potassium salt (SPA)/CsPbI_3_/Spiro/Au	[320]
	20.25	94	10224	Xenon (100 mW cm^−2^)/20–30 °C/10–15% RH/unencap	FTO/c‐TiO_2_/CsPbI_3_:PAA/P3HT/Au	[334]
	19.52	92	1400	Xenon (100 mW cm^−2^)/23 °C/20% RH/unencap	FTO/c‐TiO_2_/PCBA/CsPb_0.95_Ge_0.05_I_3_ /Spiro‐OMeTAD/Au	[324]
	16.9	90	2000	w‐LED (100 mW cm^−2^)/25 °C/20% RH/unencap.	FTO/ZnO–ZnS/m‐TiO2‐CsPbI3‐ 18‐crown‐6 ether/Spiro‐OMeTAD/Au	[292]
	9.5	90	3000	Xenon (100 mW cm^−2^)/20–30 °C/10–20% RH/unencap	FTO/c‐TiO_2_/m‐TiO_2_/CsPbI_3_ (via HPbI_3_)/carbon	[156]
	20.02	≈100	1152	Xenon (100 mW cm^−2^)/25 °C/N_2_/unencap.	FTO/P3CT‐N/CsPbI_3_/1,3‐propylene diamine (PDA)/PCBM/BCP/Ag	[336]
		90	1152	Xenon (100 mW cm^−2^)/25 °C/30–35% RH/unencap.		
	21.38	92	2880	w‐LED (100 mW cm^−2^)/25 °C/N_2_/unencap.	ITO/PEDOT:PSS/Me‐4PACz/CsPbI_3_:Me‐4PACz:EAL/PCBM/C60/BCP/Ag	[162]
	20.0	95 (*J*–*V* before and after storage)	7200	Xenon (100 mW cm^−2^)/25 °C/N_2_/unencap.	ITO/P3CT‐N/CsPbI_3_:DPhTA/PCBM/C60/TPBI/Ag	[337]
	12.04	100	1560	Xenon (100 mW cm^−2^)/25 °C/N.M/encap.	FTO/c‐TiO_2_/m‐TiO2/CsPb_0.85_In_0.15_I_1.45_Br_1.55_ (CsPbI_3_:Br:InI_3_)/Carbon	[338]
	21.4	93.1	1000	Xenon (100 mW cm^−2^)/25 °C/20–25% RH/unencap.	FTO/NiO*_x_*/MeO‐2PACz/CsPbI_3−_ *_x_* Br*_x_*:Yb^3+^ /PCBM/BCP/Ag	[339]
	22.07	92.8	600	Xenon (100 mW cm^−2^)/25 °C/15–25% RH/unencap.	ITO/SnO_2_/CsPbI_2.85_Br_0.15_/ODADI/Spiro‐OMeTAD/Au	[18]
	20.6	90	1000	Xenon (100 mW cm^−2^)/25 °C/15–25% RH/unencap.	FTO)/NiO*_x_*/CsPbI_3−_ *_x_* Br*_x_*:MMI/PCBM/BCP/Ag	[258]
	18.06	81	1000	Xenon (100 mW cm^−2^)/25 °C/20–25% RH/unencap.	ITO/SnO_2_/CsPbI_3−_ *_x_* Br*_x_*:GABr/Spiro‐OMeTAD/Au	[340]
		90	1000	Xenon (100 mW cm^−2^)/25 °C/N_2_/unencap.	ITO/SnO_2_/CsPbI_3−_ *_x_* Br*_x_*:GABr/Spiro‐OMeTAD/Au	
	17.02	80	1000	Xenon (100 mW cm^−2^)/25 °C/30% RH/unencap.	FTO/NiO*_x_*/Cr‐MOF‐ CsPbI_2_Br/ZnO/C_60_/Ag	[341]
	15.92	91.7	1300	w‐LED (100 mW cm^−2^)/25 °C/20% RH/unencap.	ITO/P3CT‐N/CsPbI_2_Br:FABr/PCBM/C60/BCP/Ag	[342]
	16.58	90	4000	w‐LED (100 mW cm^−2^)/25 °C/20–30% RH/encap.	ITO/SnO_2_/CsPbI_2_Br/oleylammonium iodide (OLAI) PTAA/Au	[343]
	14.31	92	2880	w‐LED (100 mW cm^−2^)/25 °C/N_2_/unencap.	ITO/SnO_2_/CsPbI_2_Br (solvent engineering)/PTAA/Au	[344]
	16.2	98.9	1200	Xenon (100 mW cm^−2^)/25 °C/N_2_/unencap.	ITO/SnO_2_/ZnO/CsPbI_2_Br:BMIMPF_6_/D‐PTAA/MoO_3_/Ag	[345]
		88.6	1000	Xenon (100 mW cm^−2^)/25 °C/30% RH/unencap.		
	14.37	96.4	1992	Xenon (100 mW cm^−2^)/25 °C/N_2_/unencap.	ITO/NiO*_x_*/CsPbI_2_Br:4‐trifluoromethyl phenylammonium iodide (CFPA)/PCBM/BCP/Ag	[346]
	10.65	90	2640	Xenon (100 mW cm^−2^)/25 °C/5% RH/unencap.	FTO/*c*‐TiO_2_/CsPbIBr_2_‐S‐benzylisothiourea hydrochloride (SBTCl)/carbon	[347]
	10.95	90	1680	Xenon (100 mW cm^−2^)/25 °C/10% RH/unencap.	FTO/c‐TiO_2_/CsPbIBr_2_ /(NiCo)_1−_ *y*Fe*y*O*x/*Graphen oxide/Carbon	[348]
	10.4	>100	1000	Xenon (100 mW cm^−2^)/25 °C/<20% RH/unencap.	FTO/c‐TiO_2_/CsPbIBr_2_‐0.50% Cu/Spiro‐OMeTAD/Ag	[349]
	10.61	98	1920	Xenon (100 mW cm^−2^)/25 °C/5% RH/unencap.	FTO/*c*‐TiO_2_/CsPbIBr_2_:polyurethane (PU)/carbon	[350]
	11.33	100	2600	Xenon (100 mW cm^−2^)/25 °C/N.M/encap.	FTO/c‐TiO_2_/CsPb_0.9_Sn_0.1_IBr_2_ /Carbon	[351]
	11.08	≈100	2400	w‐LED (100 mW cm^−2^)/25 °C/80% RH/unencap.	FTO/SnO_2_‐TiO*_x_* Cl_4‐2x_ /CsPbBr3 Ti_3_C_2_Cl_x_ /Ti_3_C_2_Cl*_x_* /Carbon	[352]
	10.65	80	2880	Xenon (100 mW cm^−2^)/25 °C/80% RH/unencap.	FTO/SnO_2_‐TiO*_x_* Cl_4‐2x_ /WS_2_ /CsPbBr3/Carbon	[353]
	10.75	90	1440	Xenon (100 mW cm^−2^)/25 °C/80% RH/unencap.	FTO/L‐TiO_2_ nanocrystals/CsPbBr_3_:OCH_3_ /carbon	[354]
	9.72	87	3120	Xenon (100 mW cm^−2^)/25 °C/90% RH/unencap.	FTO/c‐TiO_2_/m‐TiO_2_/CsPbBr_3_/Graphene QDs/Carbon	[355]
	10.45	≈92	3600	Xenon (100 mW cm^−2^)/25 °C/80% RH/unencap.	FTO/c‐TiO_2_/m‐TiO_2_/CsPbBr_3_ (evaporation assisted deposition)/Mns/Carbon	[356]
	7.37	100	1500	Xenon (100 mW cm^−2^)/25 °C/30–35% RH/unencap.	FTO/c‐TiO_2_/m‐TiO_2_/CsPbBr_3_ (CsAc + MAAc)/Carbon	[357]
	6.1	97	4800	Xenon (100 mW cm^−2^)/20–30 °C/25–85% RH/unencap.	FTO/c‐TiO_2_/m‐TiO_2_/CsPbBr_3_ (CsBr‐IPA treatment)/Carbon	[358]
	6.7	≈100	2640	Xenon (100 mW cm^−2^)/25 °C/90–95% RH/unencap.	FTO/c‐TiO_2_/m‐TiO_2_/Cs_0.91_Rb_0.09_PbBr_3_ /Carbon	[17]
Light (MPP)	20.25	93	570	w‐LED (100 mW cm^−2^)/30–40 °C/10–25% RH/unencap	FTO/c‐TiO_2_/CsPbI_3_:PAA/P3HT/Au	[334]
	21.14	95	500	w‐LED (100 mW cm^−2^)/25 °C/N_2_/unencap.	FTO/TiO_2_/CsPbI_3_:DMAAc/Spiro‐OMeTAD/Au	[253]
	18.7	95	2000	w‐LED (100 mW cm^−2^)/25 °C/N_2_/unencap.	FTO/c‐TiO_2_/PCBA/CsPbI_3_/spiro‐OMeTAD/Au	[359]
	19.52	≈100	3000	w‐LED (100 mW cm^−2^)/25 °C/N_2_/unencap.	FTO/TiO_2_/PCBA/CsPb_0.95_Ge_0.05_I_3_ /Spiro‐OMeTAD/Au	[324]
	20.17	95	1800	w‐LED (100 mW cm^−2^)/25 °C/N_2_/unencap.	ITO/P3CTN/CsPbI_3_ with Pb(OAc)_2_ precursor/PCBM/C60/BCP/Ag	[244]
	20.0	95	1200	w‐L (100 mW cm^−2^)/45 °C/N_2_/unencap.	ITO/P3CT‐N/CsPbI_3_:DPhTA/PCBM/C60/TPBI/Ag	[337]
	21.4	90.1	1260	w‐LED (100 mW cm^−2^)/25 °C/20–25% RH/unencap.	FTO/NiO*_x_*/MeO‐2PACz/CsPbI_3−_ *_x_* Br*_x_*:Yb^3+^ /PCBM/BCP/Ag	[17]
	18.64	94	1000	w‐LED (100 mW cm^−2^)/25 °C/N_2_/unencap.	ITO/SnO_2_/LiF/CsPbI_3‐x_Br_x_:PbCl_2_ /Spiro‐OMeTAD/Au	[30]
	21.02	86	400	w‐LED (100 mW cm^−2^)/40 °C/N_2_/unencap.	ITO/SnO_2_/CsPbI_3−_ *_x_* Br*_x_*/CsF/Spiro‐OMeTAD/Au	[360]
	20.6	91	200	w‐LED (100 mW cm^−2^)/25 °C/N_2_/unencap.	FTO)/NiO*_x_*/CsPbI_3−_ *_x_* Br*_x_*:MMI/PCBM/BCP/Ag	[340]
	12.4	90	250	w‐LED (100 mW cm^−2^)/N.M/N_2_/unencap.	FTO/c‐TiO_2_/CsPbI_3−_ *_x_* Br*_x_*:halide ratio/Carbon	[361]
	16.58	90	1000	w‐LED (90 ± 5 mW cm^−2^)/40 °C/20–30% RH/encap.	ITO/SnO_2_/CsPbI_2_Br/oleylammonium iodide (OLAI) PTAA/Au	[343]
	16.24	88	1000	w‐LED (100 mW cm^−2^)/45 °C/40% RH/encap.	FTO/c‐TiO_2_/CsPbIBr_2_‐GdCl_3_ /Spiro‐OMeTAD/Ag	[362]
	15.04	95.2	480	w‐LED (100 mW cm^−2^)/45 °C/N_2_/unencap.	FTO/NiMgLiO/CsPbI_2_Br/CdS/Au	[363]
	15.18	100	600	w‐LED (100 mW cm^−2^)/25 °C/N_2_/unencap.	ITO/NiO*_x_*/CsPbI_2_Br/Ti_0.9_Sn_0.1_O_2_ /Ag	[266]
	16.4	81	1000	w‐LED (100 mW cm^−2^)/25 °C/N_2_/unencap.	ITO/c‐TiO_2_/SnO_2_/CsPbI_2_Br‐PT/PBDB‐T/MoO_3_/Ag	[364]
	10.61	56	28	w‐LED (100 mW cm^−2^)/25 °C/50% RH/unencap.	FTO/*c*‐TiO_2_/CsPbIBr_2_:polyurethane (PU)/carbon	[350]
	10.75	100	22	Xenon (100 mW cm^−2^)/25 °C/50% RH/unencap.	FTO/L‐TiO_2_ nanocrystals/CsPbBr_3_:OCH_3_ /carbon	[354]
	10.60	40	200	w‐LED(100 mW cm^−2^)/25 °C/50% RH/unencap.	FTO/SQE36/CsPbBr_3_/CsSnBr_3_ QDs/carbon	[365]
	8.32	99	1000	w‐LED (100 mW cm^−2^)/25 °C/N.M/unencap.	FTO/c‐TiO_2_/ZrO_2_/Carbon/CsPbBr_3_:N‐methylformamide (NMF) solvent/Carbon	[366]
	10.67	96	600	w‐LED (100 mW cm^−2^)/25 °C/N.M/unencap.	FTO/c‐TiO_2_/m‐TiO_2_/CsPbBr_3_/ReSe_2_ /carbon	[368]
Thermal	16.9	91	1000	w‐LED (100 mW cm^−2^)/85 °C/N_2_/unencap.	FTO/ZnO–ZnS/m‐TiO2‐CsPbI3‐18‐crown‐6 ether/Spiro‐OMeTAD/Au	[292]
	21.0	94	200	w‐LED (100 mW cm^−2^)/60 °C/N_2_/unencap.	FTO/TiO_2_/CsPbI_3_:PTAI/Spiro‐OMeTAD/Au	[321]
	9.39	100	200	Xenon (100 mW cm^−2^)/85 °C/10–20% RH/unencap.	FTO/c‐TiO_2_/m‐TiO_2_/CsPbI_3_ :PZDI_2_ /carbon	[368]
	10.74	90	500	Xenon (100 mW cm^−2^)/60 °C/N_2_/unencap.	FTO/c‐TiO_2_/m‐TiO/CsPbI_3_:PVP/Spiro‐OMeTAD/Au	[369]
	20.0	83.1	250	Xenon (100 mW cm^−2^)/65 °C/N_2_/unencap.	ITO/P3CT‐N/CsPbI_3_:DPhTA/PCBM/C60/TPBI/Ag	[337]
	21.8	83	250	Xenon (100 mW cm^−2^)/65 °C/N_2_/unencap.	FTO/TiO_2_/CsPbI_3−_ *_x_* Br*_x_*/Spiro‐OMeTAD:TA/Au	[16]
	21.35	84.3	120	Xenon (100 mW cm^−2^)/65 °C/N_2_/unencap.	FTO/TiO_2_/CsPbI_3_‐xBrx/TFA/Spiro‐OMeTAD/Au	[32]
	21.75	89.3	120	w‐LED (100 mW cm^−2^)/85 °C/N_2_/unencap.	FTO/TiO_2_/CsPbI_3−_ *_x_* Br*_x_*/BMBC/Spiro‐OMeTAD/Au	[306]
	21.4	86.6	350	w‐LED (100 mW cm^−2^)/65 °C/N_2_/unencap.	FTO/NiO*_x_*/MeO‐2PACz/CsPbI_3−_ *_x_* Br*_x_*: Yb^3+^ /PCBM/BCP/Ag	[17]
	14.0	92.1	1000	w‐LED (100 mW cm^−2^)/85 °C (storage)/N_2_/encap.	FTO/Li, Mg doped NiOx (NiMgLiO)/CsPbI_2_Br/C–MO_X_ /Ag	[370]
	15.59	88	720	Xenon (100 mW cm^−2^)/85 °C/N_2_/unencap.	FTO/c‐TiO_2_/CsPbIBr_2_‐4‐GBACl/Spiro‐OMeTAD/Ag	[371]
	16.1	84	500	Xenon (100 mW cm^−2^)/85 °C/<10% RH/unencap.	FTO/c‐TiO_2_/1D‐3D CsPbI_2_Br/Spiro‐OMeTAD/Au	[372]
		92	500	Xenon (100 mW cm^−2^)/85 °C/N_2_/unencap.		
	17.02	80	1000	Xenon (100 mW cm^−2^)/85 °C (storage)/<10% RH/unencap.	FTO/NiO*_x_*/Cr‐MOF‐ CsPbI_2_Br/ZnO/C_60_/Ag	[341]
	15.69	95	1600	Xenon (100 mW cm^−2^)/85 °C (storage)/40–50% RH/unencap.	FTO/c‐TiO_2_/CsPbI2Br:InCl_3_ /P3HT/Au	[373]
	15.18	85	200	w‐LED (100 mW cm^−2^)/85 °C/N_2_/unencap.	ITO/NiO*_x_*/CsPbI_2_Br/Ti_0.9_Sn_0.1_O_2_ /Ag	[266]
	10.4	75	1000	Xenon (100 mW cm^−2^)/90 °C/<30% RH/unencap.	FTO/c‐TiO_2_/CsPbIBr_2_‐0.50% Cu/Spiro‐OMeTAD/Au	[349]
	10.61	102	840	Xenon (100 mW cm^−2^)/85 °C/20% RH/unencap.	FTO/*c*‐TiO_2_/CsPbIBr_2_:polyurethane (PU)/carbon	[350]
	10.51	81	400	Xenon (100 mW cm^−2^)/85 °C/N_2_/unencap.	FTO/c‐TiO_2_/CsPb_0.99_Zn_0.01_IBr_2_ /Spiro‐OMeTAD/Ag	[374]
	10.94	96	1080	Xenon (100 mW cm^−2^)/25 °C/N_2_/unencap.	FTO/c‐TiO_2_/CsPbIBr_2_:temperature‐dependent halide exchange/Carbon	[375]
	11.33	100	336	Xenon (100 mW cm^−2^)/100 °C/N_2_/unencap.	FTO/c‐TiO_2_/CsPb_0.9_Sn_0.1_IBr_2_ /Carbon	[351]
	10.71	88	720	w‐LED (100 mW cm^−2^)/85 °C/85% RH/unencap.	FTO/TiO_2_/EMImCl‐CsPbBr_3_/Carbon	[34]
	10.31	94	720	w‐LED (100 mW cm^−2^)/85 °C/85% RH/unencap.	FTO/c‐TiO_2_/m‐TiO_2_/DAP/CsPbBr_3_/carbon	[376]
	11.08	≈100	720	w‐LED (100 mW cm^−2^)/85 °C/40% RH/unencap.	FTO/SnO2‐TiO_x_Cl_4‐2x_ /CsPbBr_3_ Ti_3_C_2_Cl_x_ /Ti_3_C_2_Cl_x_ /Carbon	[352]
	10.67	94	1200	w‐LED (100 mW cm^−2^)/85 °C (storage)/0% RH/unencap.	FTO/c‐TiO_2_/m‐TiO_2_/CsPbBr_3_/ReSe_2_ /carbon	[367]
	10.45	80	2400	Xenon (100 mW cm^−2^)/85 °C/80% RH/unencap.	FTO/c‐TiO_2_/m‐TiO_2_/CsPbBr_3_ (evaporation assisted deposition)/Mns/Carbon	[356]
Thermal cycling	16.2	99.5	200 cycles	Xenon (100 mW cm^−2^)/25–100 °C/N_2_/unencap.	ITO/SnO_2_/ZnO/CsPbI_2_Br:BMIMPF_6_/D‐PTAA/MoO_3_/Ag	[345]

^a)^ Best PCE reported in literature, devices selected for long‐term stability do not essentially have the besst PCE. The novelty or strategy used in the papers is marked in green in the architectural column.

### 7.1 Precursor and Solvent Engineering

Sun et al. discovered that substituting Pb(OAc)_2_ with PbI_2_ in a precursor containing DMAI enhanced the stability and performance of the material by modulating its crystallization dynamics.^[^ ^244^ ^]^ The reactions between acetate and dimethylammonium in the wet film facilitated internal solvent annealing, generating solvent vapors for self‐solvent annealing and accelerating the removal of dimethylammonium additives. To evaluate the impact of the substitution approach on device stability, films were aged at 40% RH, observing a rapid phase transition in the reference films but no color change associated with the phase transition in the PbAc_2_‐tailored films owing to the promoted crystallization of CsPbI_3_. The enhanced stability was reflected in the operational stability of CsPbI_3_ encapsulated solar devices, which maintained 95% of their initial efficiency after continuous operation at the MPP for 1800 h under 1 sun conditions. Chen et al. grew high‐crystal quality α‐CsPbI_3_ films using a PbI_2_·NMP‐assisted growth technique and applying 18‐crown‐6 ether as a defect passivator to enhance moisture endurance. The resulting stable α‐CsPbI_3_ PSCs maintained ≈91% of their initial PCE for over 1000 h at 85 °C in a low‐oxygen (nitrogen) environment (ISOS‐D) and ≈90% for up to 2000 h at room temperature (25 °C) with 20% RH (ISOS‐D‐1), without requiring encapsulation.^[^ ^292^ ^]^ Additionally, they achieved stability, retaining ≈95% of their original efficiency for over 500 h under illumination with 60% RH and encapsulation (ISOS‐L‐1).

Several studies have transformed DMAPbI_3_ into CsPbI_3_ to enhance the stability of the black phase. This process often involves incorporating DMAI or HI along with the precursors, demonstrating an effective approach for achieving this stabilization. While the formation of DMAPbI_3_ facilitates the production of black‐phase CsPbI_3_ at low temperatures, there have been varying opinions on the complete removal of DMA^+^ for device performance, as discussed in previous sessions. Li et al. reaffirmed the complete removal of DMA^+^ by employing hydrogen bonding to aid in removing DMA^+^ by incorporating polyacrylic acid (PAA) in the precursor solution. As a result, the constructed devices exhibited exceptional shelf‐life stability, retaining over 94% of the initial efficiency for 10 224 h during storage in dark and low‐humidity (<15% RH) conditions.^[^ ^348^ ^]^ In a study, the thermally accelerated degradation of encapsulated CsPbI_3_ PSCs under constant illumination in 35–110 °C and ≈65% RH (ISOS‐L‐2 and ISOS‐L‐3) was investigated to assess the commercial potential of inorganic PSCs. The degradation of PCE followed an Arrhenius temperature dependence that was mitigated by a 2D Cs_2_PbI_2_Cl_2_ layer on CsPbI_3_.^[^ ^320^ ^]^ The results demonstrated a *T*_80_ of over 2100 h at 110 °C and 65% RH. With an experimentally determined acceleration factor of 24.2 ± 3.5, the extrapolated *T*_80_ was 5.1 ± 0.7 × 10^4^ h, which translated to more than 5 years of continuous operation at 35 °C.

### 7.2 Additive and Composition Engineering

Despite the known instability of Cs–Ge perovskites due to oxidation,^[^ ^321^ ^]^ incorporating a small amount of GeI_2_ into CsPbI_3_ to create CsPb_0.95_Ge_0.05_I_3_, along with adding a GeO_2_ passivation layer, results in the development of devices that can retain 92% of their performance after 1400 h of storage at room temperature and 20% RH (ISOS‐D‐1).^[^ ^322^ ^]^ Furthermore, the photostability of the unencapsulated devices under MPP tracking under an LED in N_2_ for 3000 h results in a PCE higher than the initial value (ISOS‐L‐1). The surface treatment of CsPbI_3_ films has shown substantial potential for enhancing their stability. Thin functionalization layers containing organic cations with tailored hydrophobic groups act as a barrier to moisture ingress, thereby protecting the phase stability of inorganic perovskites.^[^ ^7^, ^323^ ^]^ For example, FBTH‐I‐treated unencapsulated CsPbI_3_ solar cells demonstrated 92.6% retention of initial PCE (21.41%) after 900 h storage in 25% RH (ISOS‐D‐1).^[^ ^245^ ^]^ The introduction of another organic additive, 4‐thioureidobenzoic acid, ensured 1000 h of stability, retaining 95.6% of initial PCE for the unencapsulated devices at 25–30 °C and 30–40% RH (ISOS‐L‐1).^[^ ^324^ ^]^

The use of guanidinium (GA) and glycocyamine as additives for CsPb(I_2.85_Br_0.149_Cl_0.001_) significantly enhances phase stability and minimizes traps, resulting in unencapsulated devices shelf‐life (*T*_80_) of 1440 h at 10–20% RH and 20 °C. Similarly, the introduction of an ionic Lewis base additive carboxyethylisothiuronium chloride (ATP) in CsPbI_2.85_Br_0.15_ results in devices retaining 96.1% of the initial PCE at 25–30 °C and 30–40% RH after 1000 h without encapsulation. The use of 4‐polyamine (4‐PA) to passivate CsPbI_2.85_Br_0.15_ has led to unencapsulated devices exhibiting 3000 h of storage stability at 25% RH while retaining 94% of their performance (ISOS‐D‐1). Wu et al. recently introduced a new approach involving the use of a rotatable structure of trimethylamine Pb halide (TrMAPbX_3_ (X = Br^−^ or I^−^)) in γ‐CsPbI*_x_* Br_3−_ *_x_* to effectively address thermally gathered defects and achieve improved thermal stability.^[^ ^325^ ^]^ The unencapsulated devices demonstrated over 84% stability retention at 85 °C in N_2_ over 192 h (ISOS‐L) and 91% when stored in 25% RH at 25 °C for 3055 h (ISOS‐D‐1).

Similarly, including CaCl_2_ in the CsPbI_2_Br devices allowed them to maintain ≈90% of their original efficiency after 1080 h of storage and testing in an environment with 25% RH (ISOS‐D‐1).^[^ ^326^ ^]^ Researchers have fabricated C‐based CsPbIBr_2_ devices that maintain ≈90% of their initial performance after being stored for 60 d (1440 h) at 25 °C and 45% RH.^[^ ^327^ ^]^ Furthermore, the devices retain ≈97% performance after being stored for 7 d (168 h) at 85 °C with a shallow water content.

### 7.3 Passivation or Surface Modification

Further, ionic liquids have been widely studied for their ability to improve long‐term stability, and various groups have achieved impressive results.^[^ ^141^, ^179^, ^182^, ^328^ ^]^ Du et al. introduced 1‐ethyl‐3‐methylimidazolium hydrogen sulfate (EMIMHSO_4_) into CsPbI_3_ perovskites.^[^ ^179^ ^]^ The resulting EMIMHSO_4_‐modified devices achieved 20.01% and maintained 95% of the initial PCE for 1000 h after storing at 25% ± 5% RH and 25 ± 5 °C (ambient) without encapsulation (ISOS‐D‐1). Similarly, Rui et al. incorporated *n*‐butylammonium hexafluorophosphate (TBAPF_6_) to enhance stability, maintaining over 70% of the initial PCE after storing for 168 h in RH 30% ± 5% (ISOS‐D‐1) for encapsulated devices and over 1600 h in the glovebox with a loss of less than 10% of the initial PCE.^[^ ^241^ ^]^ The use of tetrabutylammonium cations (TBA^+^), with their strong intercalation ability and F‐based molecules with hydrophobic properties that inhibit I vacancies and prevent moisture, has significantly improved film stability.

Similarly, Pu et al. utilized 1‐viny‐3‐propionate ethyl imidazolium chloride ([PEVIM]Cl) and retained 91% of the original PCE for C‐based CsPbI_2_Br‐PSCs kept at 85 °C in N_2_ for 360 h (ISOS‐L).^[^ ^329^ ^]^ After 1440 h of storage in air at ≈25% RH (ISOS‐D‐1), the CsPbI_2_Br solar cells treated with BMMIMBF4 retained 94.4% of their initial PCE, demonstrating the long‐term stability of the treatment.^[^ ^330^ ^]^

From a structural stability standpoint, CsPbBr_3_ has the highest Goldschmidt tolerance factor (0.86) among all inorganic Pb‐based perovskites, which stabilizes its photoactive phase at room temperature. Passivating the CsPbBr_3_ with an ionic liquid (such as EMImCl) and constructing C‐based HTL‐free CsPbBr_3_ solar cells can maintain 80% of its initial PCE after 120 d at 25 °C, 80% RH, and 90% after 30 d at 85 °C, 85% RH without any encapsulation.^[^ ^331^ ^]^ C‐based HTL‐free devices show no decrease in performance when stored at 90–95% RH at room temperature for over three months.^[^ ^17^ ^]^ The devices demonstrate 840 h of thermal stability when tested at 100 °C and do not degrade in extreme temperatures ranging from −22 to 100 °C for 80 h. Zhang et al. introduced 1‐butyl‐2,3‐dimethylimidazolium chloride ([BMMIm]Cl) and retained 98.5% PCE in 70% RH at 20 °C for over 720 h without encapsulation (ISOS‐D).^[^ ^332^ ^]^ Additionally, the [BMMIm]Cl‐passivated device demonstrates light‐thermal stability under continuous illumination for 720 h in a moisture‐free (0% RH) environment at 80 °C (ISOS‐L).

Through the use of thermally stable phenyltrimethylammonium iodide (PTAI) for surface passivation to form lower‐dimensional perovskites in CsPbI_3_ crystal grains,^[^ ^45^ ^]^ researchers have achieved shelf‐life stability (ISOS‐D‐1) of over 2000 h, maintaining 83% of the initial PCE under low humidity (10–15%) at room temperature. It is important to note that these devices were tested without encapsulation.

The stability advances show that CsPbX_3_ perovskites have demonstrated greater thermal and photostability than hybrid PSCs. However, the stability in highly humid conditions, particularly for CsPbI_3_ solar cells, is still a cause for concern (Figure 10 and Table 3). It has been observed that bromine‐rich CsPbX_3_ compositions exhibit good moisture and thermal stability without device encapsulation. Yet, bromine‐rich compositions appear to have relatively lower photostability than their iodine counterparts, which calls for further investigation of the material.

While significant progress has been made in enhancing the lifetime of inorganic PSCs, accurately comparing reported stabilities across various studies remains challenging due to the absence of unified evaluation criteria as researchers overlook assessment protocols. Although impressive stabilities have been demonstrated under controlled conditions through additive or surface engineering, emphasis on real‐world protocols such as outdoor stability (ISOS‐O), thermal cycling (ISOS‐T), and light cycling (ISOS‐LC) are crucial for inorganic PSCs. Furthermore, indications predict fluctuations in the efficiency and stability of PSCs when exposed to cyclic light conditions, such as real‐time outdoor settings, instead of constant illumination.^[^ ^377^, ^378^ ^]^ Nonetheless, given the rapid pace of development in this field, more stringent, commercially relevant stability studies are expected in the future.

## 8 Challenges and Opportunities

The PCE of inorganic perovskites has steadily increased, reaching over 20%. However, enormous challenges hinder the commercialization of this technology. One major issue involves the vulnerability of inorganic lead‐halide perovskite films to spontaneous phase transitions, which limits their low‐cost fabrication and operational stability.

In addition, defects in inorganic perovskites are critical challenges affecting the efficiency and stability of PSCs. Studies have demonstrated that defects, particularly point defects, play a significant role in determining PSC performance. Nonradiative recombination induced by defects is more significant on the surface of CsPbI_3_ perovskites than in bulk, which limits device performance and stability. Studies have identified I‐vacancies and structural defects as the primary reasons for the structural collapse of the nonphotoactive δ‐phase from the γ‐phase in CsPbI_3_ perovskites.

Additionally, significant progress has been made in Br‐rich inorganic PSCs, with extensive efforts demonstrating the operational stability of CsPbBr_3_/C devices under harsh thermal moisture conditions for over half a year without encapsulation. Despite these efforts, the highest PCE reported for inorganic CsPbBr_3_ solar cells is lower than the Shockley–Queisser limit of 16.4% due to the wide bandgap of CsPbBr_3_ solar cells, which lose a significant part of their electromagnetic spectrum, resulting in a low short‐circuit current. Additionally, the trap states and unoptimized interfacial layers contribute to significant voltage loss (>0.6 V) compared to high‐efficiency organic–inorganic hybrid perovskite, which is already lower than <0.4 V.^[^ ^379^, ^380^, ^381^ ^]^ Furthermore, using polar solvents such as DMSO or water to dissolve high‐Cs and high‐Br content precursors causes solvent penetration through the interconnection layer, potentially damaging the other interfacial layers. Addressing the issue is critical for ensuring the high efficiency and long‐term stability of inorganic PSCs.

For low‐cost and large‐scale manufacturing, addressing high‐temperature and prolonged annealing of inorganic perovskites is critical for facilitating the path toward commercialization. Efforts such as precursor engineering using DMAI or HI, which leads to the formation of DMAPbI_3_, can control crystallization and obtain black‐phase CsPbX_3_ perovskites at low temperatures. Additionally, strategies such as the additive engineering of A‐cations or solvent engineering using orthogonal solvent mixing have enabled lower temperature processing and enhancement of the intrinsic stability of CsPbX_3_ perovskites. Utilizing more environmentally friendly solvents, such as acetonitrile, ethyl acetate, and 2‐propanol, suitable for large‐scale processing remains a challenge; optimizing the fabrication of CsPbX_3_ under ambient conditions involves skillfully managing the effects of moisture. While excessive moisture can compromise the crystallinity of perovskite, resulting in the transformation to a nonphotoactive yellow phase and, therefore, to PbI_2_, a controlled amount of water at the grain boundary can enhance the quality of the film by facilitating merged grains, thereby reducing surface defects. To produce high‐quality crystalline films, annealing in air at a controlled humidity is recommended. Additionally, incorporating ionic liquids or alkyl ammonium iodide passivation strategies is crucial for mitigating moisture ingress and minimizing defects, thereby preventing environmentally induced phase transitions. The approach not only aids in adjusting crystallization and promoting the formation of dense, large‐sized grains but also contributes to enhancing long‐term stability under ambient conditions by safeguarding against excessive moisture intrusion.

Charge transport layers require further development to address efficiency loss, which is caused by interfacial recombination often because of the energy level mismatch between the perovskite and carrier transport layers. Although conventional perovskite charge‐transport layers have been extensively studied for the development of inorganic perovskite devices such as SnO_2_ and spiro‐OMeTAD, there is limited research on alternative energy‐aligned novel or modified ETLs and HTLs designed to minimize charge‐transport losses in inorganic PSCs. The development of high‐mobility, energy‐level‐matched charge transport layers presents a practical strategy for reducing the energy barriers between perovskite and transport layers, thereby enhancing the extraction and transfer of photogenerated carriers in perovskite films. Potentially, all inorganic charge‐transport layers in combination with inorganic perovskite films can also significantly improve stability. However, significant challenges persist in developing inorganic ETL and HTL with proper energy level alignment. Additionally, the current development in this approach seems to require a compromise in efficiency and low‐cost manufacturing potential as one of the inorganic ETL or HTL requires either high‐temperature processing or deposition through dry vacuum‐based techniques such as sputtering or plasma deposition.

In the field of multijunction solar cells utilizing CsPbX_3_, there is a significant opportunity for growth, as research on multiple junctions remains less developed compared to single‐junction CsPbX_3_ PSCs. Much of the existing literature emphasizes hybrid PSCs‐based four‐terminal tandem solar cells, which can be attributed to challenges such as processability, phase instability, and current matching among subcells. By focusing on resolving these issues related to processability and phase stability, particularly through advancements in additive, solvent, surface, and device engineering, we can significantly enhance the performance of both single‐junction and tandem solar cells.

Inorganic PSC‐based research into stability and scalability lags behind that of hybrid counterparts. Encapsulation is essential for improving the durability of PSCs, as it shields them from the ingress of moisture and O_2_ that can accelerate degradation. There are limited discussions on the influence of the encapsulation materials and methods employed on the durability of the tested inorganic PSCs. Further research on the influence of encapsulation materials and methods and improvement in the encapsulation protocol would potentially improve the durability of both PSCs in general and inorganic PSCs in particular.

A limited number of studies have investigated the fabrication of inorganic PSCs through scalable techniques. However, early studies show promising progress, achieving 19% efficiency using blade coating in a noninert atmosphere. Future studies should explore research on the fabrication of inorganic PCSs through scalable deposition methods in ambient environments using low temperatures and fast annealing steps. Despite existing challenges, inorganic perovskites show outstanding promise as affordable and durable light absorbers for PV applications, given their low material and processing costs and potential for higher stability.

## Conflict of Interest

The authors declare no conflict of interest.
